# Response of the surface-active beetle community (Coleoptera) to mixed-conifer forest restoration thinning and burning treatments in northern New Mexico, USA

**DOI:** 10.1093/ee/nvag098

**Published:** 2026-09-17

**Authors:** Robert R Parmenter, Richard J Buss, Benjamin P Stout, Sarah M Hall, Orrin B Myers

**Affiliations:** Valles Caldera National Preserve, National Park Service, Jemez Springs, NM, USA; Museum of Southwestern Biology, Division of Arthropods, Department of Biology, University of New Mexico, Albuquerque, NM, USA; Valles Caldera National Preserve, National Park Service, Jemez Springs, NM, USA; Museum of Southwestern Biology, Division of Arthropods, Department of Biology, University of New Mexico, Albuquerque, NM, USA; Valles Caldera National Preserve, National Park Service, Jemez Springs, NM, USA; Valles Caldera National Preserve, National Park Service, Jemez Springs, NM, USA; Department of Family and Community Medicine, University of New Mexico, Albuquerque, NM, USA

**Keywords:** Arthropoda, forest logging, insect succession, managed fire, silviculture

## Abstract

Forests in the western United States are experiencing increasing stresses from more frequent stand-replacing fires, insect outbreaks, and droughts. To mitigate these phenomena, forest managers are increasingly employing forest fuels management operations (tree thinning and prescribed fires) to reduce fire severities and inter-tree competition for water and soil nutrients. While such forest restoration programs have proven effective, the impacts on forest arthropod communities are largely unreported. In this 6-year case study, we examined the response of a surface-active (epigeic) beetle community (Coleoptera) in a mixed-conifer forest subjected to tree thinning followed by a prescribed broadcast slash burn. We used a Before-After/Control-Impact (BACI) design to test treatment effects on the beetle community composition, diversity, richness, evenness, individual species abundances, and trophic structure. We documented 289 taxa of Coleoptera and found that treatments caused significant changes in the beetle community’s composition, with 21 common species increasing and 35 species declining in the more open post-treatment sub-canopy environment; only 3 common species were extirpated from the treatment sites, while 2 common species colonized the post-thinned/burned areas. Saproxylic species exhibited a decrease compared to controls after thinning and prescribed fire. Trophic structure species composition did not change, although numbers of individuals increased in the omnivore, fungivore, and detritivore trophic groups. Overall, our forest restoration treatments did not reach the level of impact from more severe forest disturbances (clear-cutting, or high-severity wildfires) and created a mosaic of grassy forest understory and small open meadow patches that would contribute to increased landscape arthropod diversity.

## Graphical Abstract

**Graphical Abstract:**
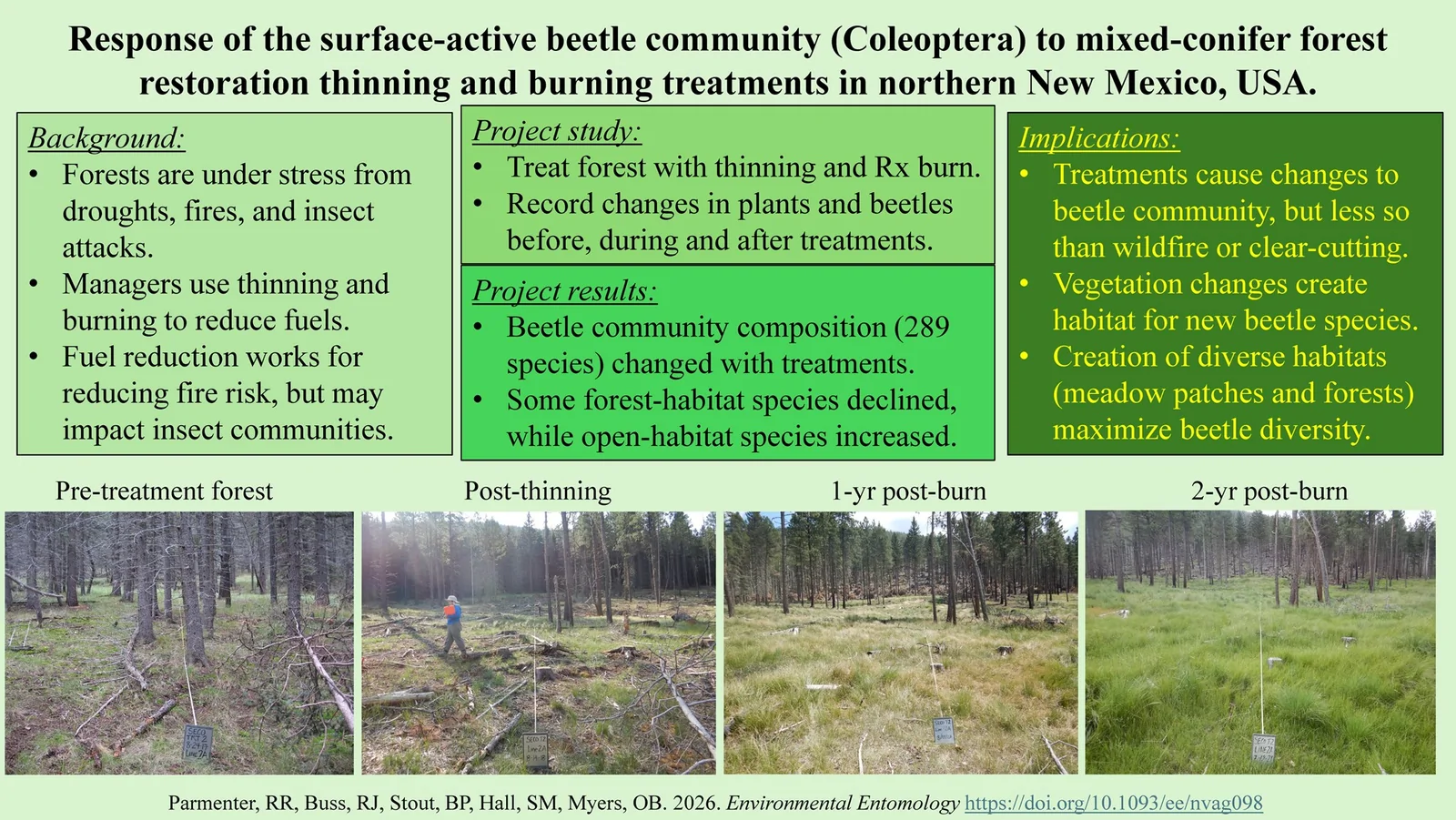

## Introduction

Forested ecosystems worldwide are under increasing stress from land-use changes and climate warming, resulting in large-scale tree mortalities from droughts, high-severity fires, insect outbreaks, and anthropogenic deforestation activities (Allen et al. 2010, Allen et al. 2015, Kelly et al. 2020, Hagmann et al. 2021, Abatzoglou et al. 2025, Parks et al. 2025, Potapov et al. 2025, Wion et al. 2026). As a result, forest managers have initiated many restoration programs, designed to increase forest resiliency and maintain forest ecosystem structure and functioning, including arthropod communities (Hessburg et al. 2021, Hua et al. 2022, Bieber et al. 2023). In fire-prone forests of the western United States, these restoration programs often involve fuels reduction operations, in the form of tree thinning, wood removal, and prescribed (planned) fires (Allen et al. 2002, Reynolds et al. 2013, Stoddard et al. 2021, Jones et al. 2025). Fuel reduction operations have been shown to be successful in reducing high-severity fires and restoring natural low-intensity fire regimes, and these approaches are being implemented with increasing frequency on the landscape (Davis et al. 2024, Loverin et al. 2024, Stephens et al. 2024, Bernal et al. 2025, Grupenhoff et al. 2025, Yackulic et al. 2025, Ziegler et al. 2025).

Forest restoration and management programs involving tree harvest and/or prescribed fire have been shown to impact arthropod communities, with more intense management disturbances (such as clear-cutting, slash/stump removals, and soil tilling) having greater impacts on arthropods than less-intrusive treatments (such as light thinning, low-intensity prescribed fires; see recent reviews by Perry and Herms 2017, Koivula and Vanha-Majamaa 2020, Mason et al. 2021, Bieber et al. 2023, Stočes and Šipoš 2025). Intensive management operations typically induce major shifts in the abundances of dominant arthropod species from forest-habitat specialists to open-habitat specialists and can often locally extirpate species requiring mature forest habitat. These shifts may result in changes in overall species *α*-diversity and even increase species richness but produce distinctly different community compositions (ibid.). At the landscape level, the mosaics of habitat patches in different seral stages can lead to increases in *β*-diversity if sufficient patches of mature forests can be maintained, thereby preserving forest habitat specialist species (ibid.).

Among the taxa of important forest arthropods are surface-active beetles (Coleoptera). Beetles comprise a taxonomically-rich community with a wide range of trophic relationships, including predators/parasitoids, herbivores/granivores, fungivores, detritivores/scavengers, and omnivores (Marshall 2018, Barclay and Bouchard 2023). Beetles are important contributors to forest ecosystem structure and functioning through trophic interactions such as herbivory (Kautz et al. 2017) and predator–prey relationships (Potapov et al. 2022), nutrient cycling and decomposition of plant litter and animal carrion (Sugiura et al. 2013, Ulyshen 2016, Siegert et al. 2024), soil disturbances and bioturbation (Wilkinson et al. 2009, Filser et al. 2016), dispersal of fungal spores (eg mycorrhizae [Naranjo-Morán et al. 2021] and tree disease pathogens [Hofstetter et al. 2015]), and providing a ­nutritious food source to many species of terrestrial and aquatic vertebrate wildlife (Potapov et al. 2022).

As such, understanding the impacts of forest fuels reduction activities on surface-active beetle communities would provide important insights into the success of forest restoration strategies. However, most of the forest management studies on beetles conducted to date in the western United States have involved higher-impact disturbances such as clear-cutting (Marra and Edmonds 1998, Clayton 2002, Heyborne et al. 2003, Halaj et al. 2008, Sultaire et al. 2021, Frank et al. 2026), post-wildfire salvage logging (Frank et al. 2026), stand-replacement wildfires (Dajoz 1998, Clayton 2002, Niwa and Peck 2002, Villa-Castillo and Wagner 2002, Chen et al. 2006, Higgins et al. 2014, Ferrenberg et al. 2019, Mott et al. 2023, Frank et al. 2026), and prescribed fires in forest stands without prior fuel reductions (Ferrenberg et al. 2006). In contrast, fewer studies have addressed less-intensive coniferous forest restoration activities of tree thinning with or without prescribed slash burning in western states; these include Willett (2001) and Apigian et al. (2006a, b) in California, Schowalter et al. (2003) in Washington, and Villa-Castillo and Wagner (2002) and Chen et al. (2006) in Arizona.

In this case study, our goal was to assess the successional dynamics of the surface-active beetle community during the first 5 years of a restoration project in a mixed-conifer forest using thinning and cool-season burning in northern New Mexico, USA. We used a Before-After/Control-Impact (BACI) experimental design to record ecological changes before, during, and after both forest mechanical thinning and prescribed broadcast-slash burning. We monitored forest stand variables, herbaceous vegetation, forest floor microhabitats, and surface-active beetle abundances during the project period in treated and control (untreated) stands. We addressed the following questions:

1. How did surface-active beetle community species diversity, richness, and evenness change through this post-treatment time period, and which (if any) species of ­beetles increased, decreased or remained unchanged in the treatment site? While our null hypothesis was “no change,” we predicted that community metrics would decrease immediately following the thinning and burning disturbances, but would then recover rapidly in the following years, while various species would increase or decrease in abundance based on habitat preferences ­(forest specialists would decrease while open-habitat specialists would increase).
2. How did the beetle community change through time (via similarity indices)? We predicted that the beetle communities of the treated and control sites would be similar prior to thinning, but that the treatments would generate observable differences through time.
3. How would the trophic structure of the beetle community change through time as a result of the forest treatments? Again, our null hypothesis was that the treatments would have no effect, but we predicted that with increases in herbaceous vegetation cover, the beetle communities would shift in favor of herbivores and omnivores in both species numbers and numbers of individuals.
4. What were the roles of logs and other habitat factors in the distribution and abundances of beetle species? We anticipated that log proximity would enhance collections of saproxylic species, but otherwise would have no effect on the abundances of other beetle species; other habitat variables should be associated with abundances of various species, but the magnitudes of these relationships were unknown prior to the study.

## Methods

### Site Description

**Fig. 1.**
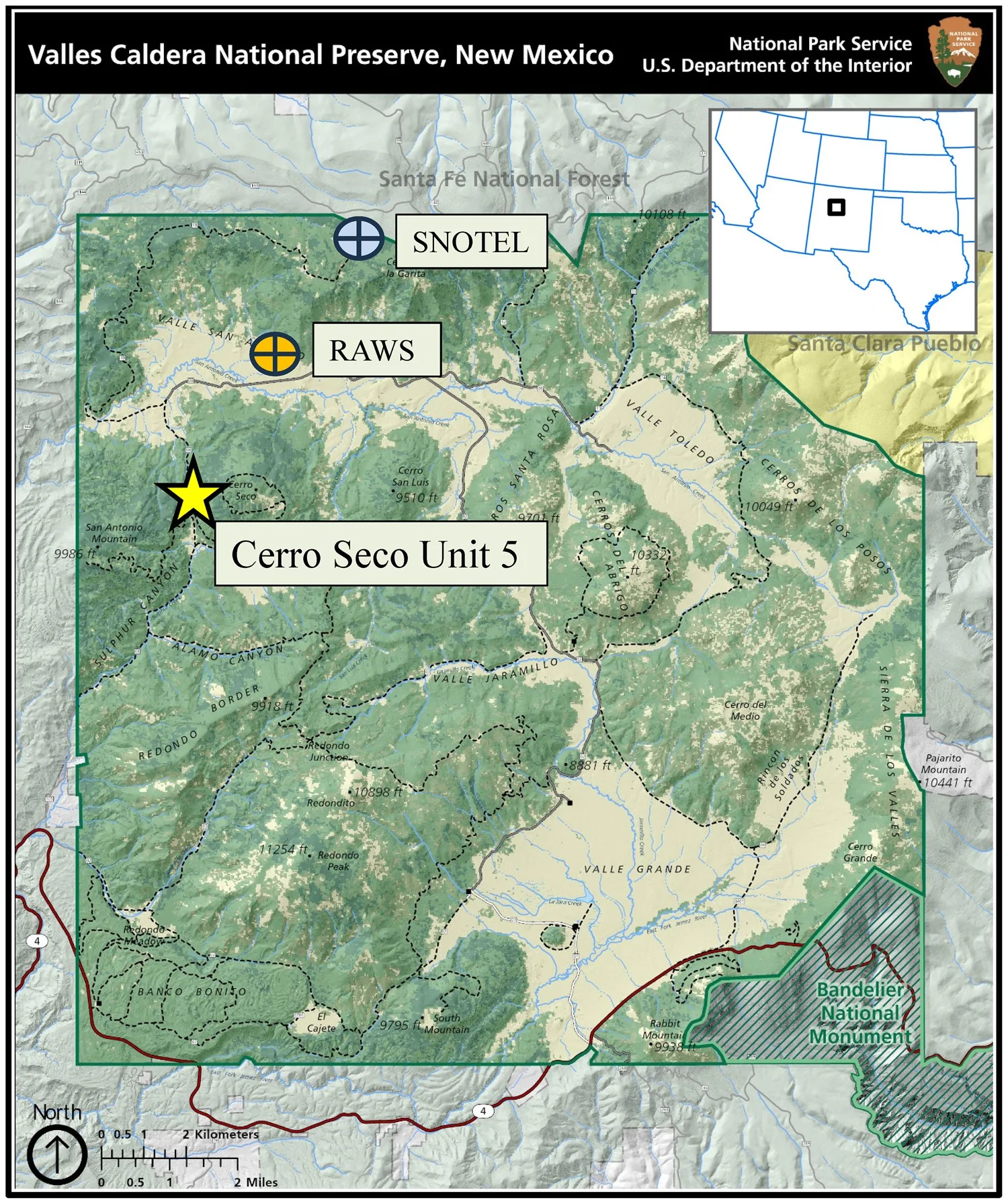
Map of Valles Caldera National Preserve, NM, showing the locations of the project area (Cerro Seco Unit 5; yellow star), the Remote Automatic Weather Station (RAWS; orange circle), and the Garita Peak Snowpack Telemetry station (SNOTEL; blue circle). Map produced by M. White, National Park Service.

We implemented the study in the National Park Service’s (NPS) Valles Caldera National Preserve (Preserve) in the Jemez Mountains of northern New Mexico (Fig. 1). The Preserve is a dormant 1.23 million-year-old volcanic caldera, within which lies an internal ring of forested rhyolite domes (Goff 2009). The project site was on one of these domes, Cerro Seco (Fig. 1; lat 35°57′11″, long 106°35′30″ elevation 2,665 m). Dominant tree species were ponderosa pine (*Pinus ponderosa* Lawson & C. Lawson), Douglas-fir (*Pseudotsuga menziesii* (Mirb.) Franco), white fir (*Abies concolor* (Gord. & Glend.) Lindl. ex Hildebr.), Engelmann spruce (*Picea engelmannii* Parry ex Engelm.), and aspen (*Populus tremuloides* Michx.). Mean annual precipitation (2003 to 2022) was 594 mm, with ∼40% falling during summer monsoons (mid-June to mid-September), and ∼50% coming in winter snowstorms. Mean monthly temperatures ranged from –4.8 °C in January to 15.4 °C in July (Valle Grande Remote Automatic Weather Station (RAWS) data, 2003 to 2022; WRCC 2022). Soils were ∼800,000 year-old alluvium pyroclasts, classified as Vitrandic Agriudolls and Hapludolls (Hibner et al. 2010).

### Forest Restoration Treatments

**Fig. 2.**
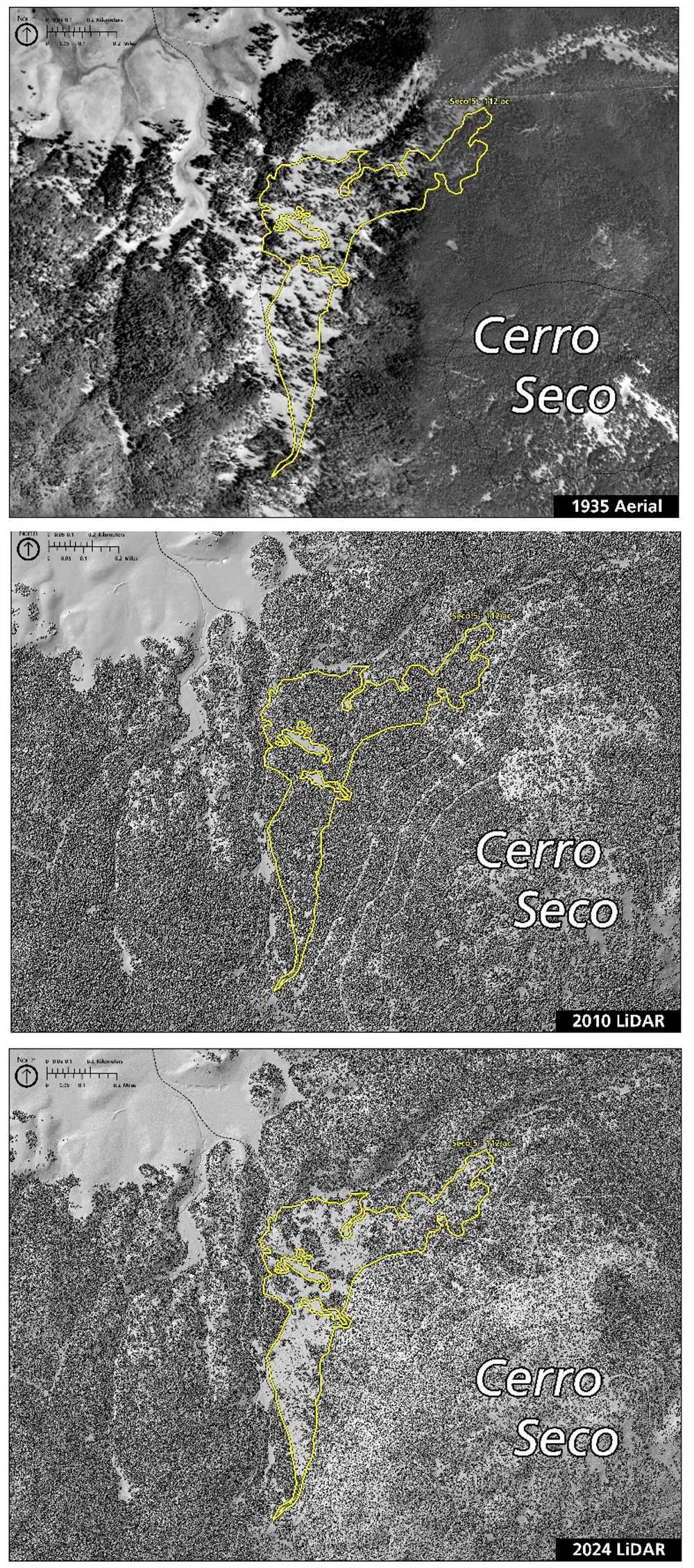
Imagery of the Cerro Seco Unit 5 project area (yellow boundary lines). Top: 1935 aerial photograph, showing open meadow mixed with scattered old growth forest patches. Middle: 2010 LiDAR (Light Detection And Ranging) image, showing infilling of second-growth forest during the 20th century. Bottom: 2024 LiDAR image, showing post-treatment conditions that approximate the forest-meadow mix of pre-1935. Note also the area on Cerro Seco to the east of Unit 5 (right side of image) that was hand-thinned during the same time period. Imagery compiled by M. White, National Park Service.

**Fig. 3.**
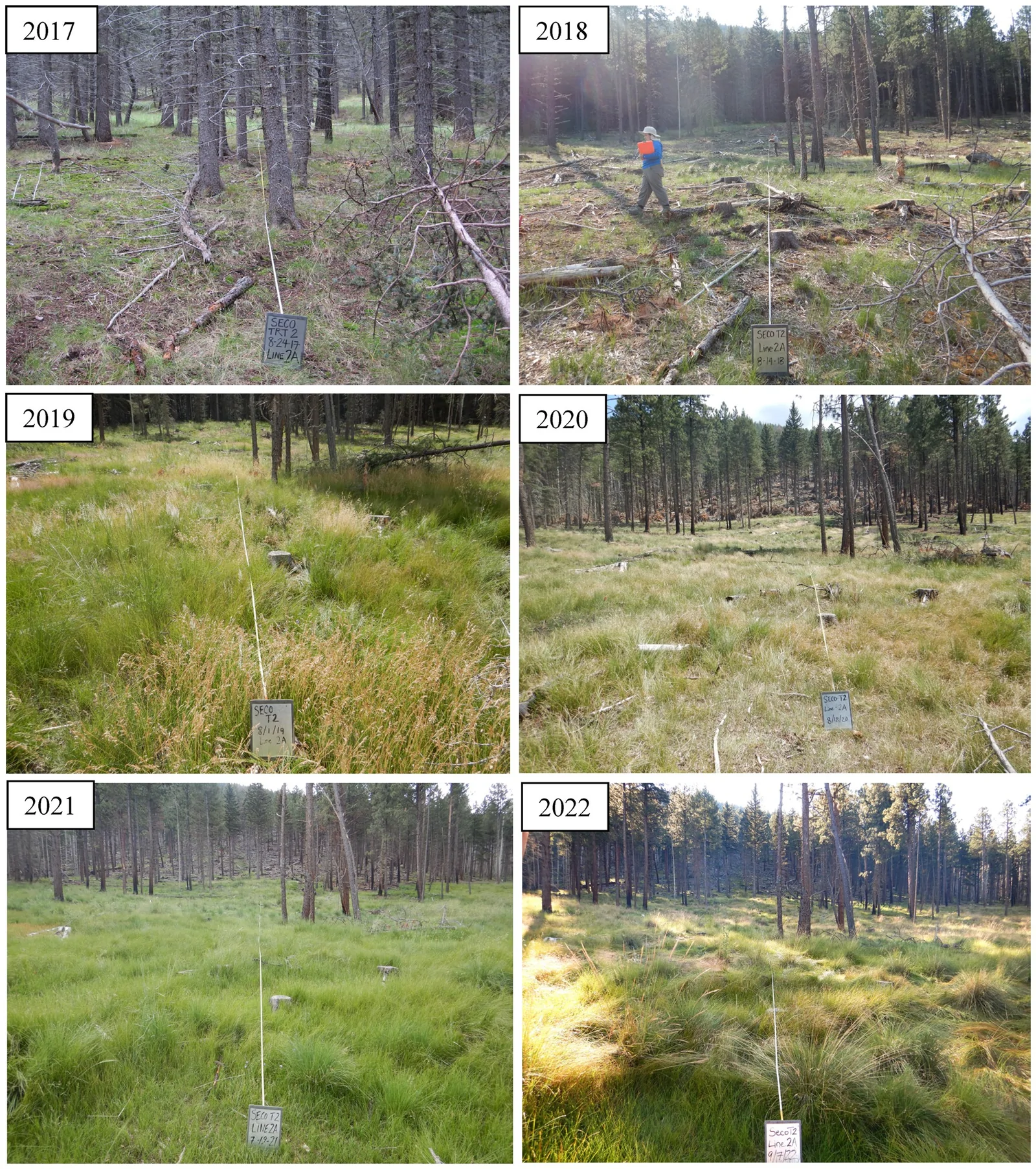
Repeat photographs of Cerro Seco Unit 5, Valles Caldera National Preserve, NM. 2017: pre-treatment second-growth forest. 2018: First-year post-thinning. 2019: Second-year post-thinning. 2020: First-year post-burn. 2021: Second-year post-burn. 2022: Third-year post-burn. Photos: NPS staff.

The study’s project site was a 45.3 ha (112 ac) second-growth mixed-conifer stand that had been selectively logged during 1980–1991 when the Preserve was under private ownership (Balmat 2004). Based on the 1935 aerial photograph (Fig. 2, top), prior to any logging or other human disturbance besides historical sheep grazing, the site had been an open meadow with scattered individuals of old-growth ponderosa pine and Douglas-fir, and patches of Engelmann spruce and white fir. With large-scale reductions in forest fires from the late 19th century to the early 21st century from livestock overgrazing (effectively eliminating fine fuels) and active fire suppression by land managers (Margolis et al. 2017, Parmenter 2022), the site had infilled with mixed-conifer forest by 2010 (tree density of >1,100 trees/ha and a nearly closed forest canopy [Fig. 2, middle]). As part of the 2010–2024 Southwest Jemez Mountains Collaborative Forest Landscape Restoration Program (CFLRP; Parmenter 2025), the restoration plan was to thin the forest, leaving all remaining ponderosa pine and Douglas-fir trees while recreating small open meadows with patches of spruce-fir forest. Forest thinning was accomplished in the winter of 2017–2018, using mechanical equipment (feller buncher, skidder, processor), and tree boles were removed to a nearby timber mill. Slash was left scattered or in small piles across the treated area (Fig. 3). Two years later (October 2019), NPS fire teams implemented a planned (prescribed) broadcast-slash burn across the site. The resulting stand structure was a close approximation of the 1935 site condition (Fig. 2, bottom). In addition, the upper slopes of Cerro Seco (adjacent to the study site) were also thinned by hand crews using chainsaws during this time, with a cut-and-pile protocol followed by pile burning; this treatment is also evident in Fig. 2 (bottom).

### Experimental Design and Beetle Sampling

We used a split-plot design with the treatment area and an adjacent untreated (control) area. We established 5 sampling sites in both areas, each of which was located next to a pre-existing downed log from the 20th-century logging activities (5 whole-plot units per treatment). Nested within each log site, we installed 6 pitfall traps for sampling surface-active arthropods. The 6 traps were arranged in 3 replicate sub-plots of pitfall traps at 5 m spacing along each log. Each sub-plot had 2 traps, one immediately adjacent to the log and another 5 m perpendicularly away from the log (see schematic layout in Appendix A, Fig. S1A and B). In this layout of a split-plot design, logs were modeled as random whole plot units and pairs of traps along each log were nested random sub-plots with log/no-log conditions in each, resulting in 30 pitfall traps per forest treatment grouping that were equally split between log/no-log placement (see schematic layout in Appendix A, Fig. S1A and B). Traps were sampled longitudinally in June, July, August, and September during years 2017—2022, which resulted in 1,440 potential spatio-temporal sampling events and 15 samples per combination of factors.

For the pitfall traps, we used plastic cups 10 cm in diameter and 14 cm deep. Cups were half-filled with propylene glycol as a preservative. A ceramic tile (23 × 23 cm), elevated 3 to 4 cm on nails, served as a cover to reduce rain and debris from entering the trap. Traps were enclosed in a steel mesh cage (5 cm × 5 cm mesh size) to prevent disturbance by elk and other large wildlife (Appendix A, Fig. S1C). Traps were open continuously each year from ∼ June 1 to ∼ Oct. 1 for 6 years: 2017 = pre-treatment; 2018 to 2019 = post-thinning; 2020 to 2022 = post-fire. Our experience has been that continuous sampling (versus periodic or episodic sampling) during the growing season produces sufficiently large sample sizes for analyses and presumably increases the probabilities of collecting rarer species during the study period. We have previously used continuous sampling successfully in arthropod studies on mine-land rehabilitation (Parmenter and MacMahon 1987), post-wildfire population responses (Parmenter et al. 2011), and post-volcanic eruption successional community changes (Parmenter et al. 2005, 2018). Samples were collected 4 times/year at the end of June, July, August, and September. If a trap was compromised by disturbance (rodent digging, flooding, etc.), the sample was discarded; this occurred 38 times out of 1,440 samples (2.6%).

Specimens were returned to the laboratory and stored in 70% ethanol until processing. Beetles were sorted, identified, and counted. We identified most taxa to species, while some were identified to genus without a species identification but were separated to “sp. #1,” “sp. #2,” etc., unless there were potentially more than 1 indistinguishable species in the genus, in which case we designated them as “spp.” (this occurred in 13 cases, all in the Staphylinidae). In addition, there was a subset of genera and species in the subfamily Aleocharinae (Staphylinidae) for which we could not confidently identify to either genus or species, and these were pooled into 1 taxon designation, “Aleocharinae spp.” Representative specimens were deposited in the Preserve’s arthropod reference collection and in the Division of Arthropods, Museum of Southwestern Biology, Department of Biology, University of New Mexico, Albuquerque, NM.

### Meteorology

To assess treatment effects on meteorological variables, we installed 4 weather stations on the study site to record air temperatures, relative humidity, precipitation, wind speed and direction, solar radiation, and soil temperatures and moisture (volumetric and matric-potential) at 10, 20, and 30 cm depth below logs and 1 to 2 m away from logs (see Appendix A, Fig. S1D; and Parmenter and Losleben (2023) for details on instrumentation). For shallow soil temperatures at 2.5 cm depth, we compiled data from a nearby RAWS; winter snowpack depth data were taken from the Natural Resources Conservation Service (NRCS) Garita Peak Snowpack Telemetry (SNOTEL) site (Fig. 1).

### Vegetation and Forest Floor Habitat Variables

Vegetation sampling and analysis methods have been described in detail in Parmenter et al. (2026), and are summarized only briefly here. We measured pre- and post-treatment forest stand characteristics utilizing standard NPS forest monitoring protocols (Suazo et al. 2013). We recorded measurements of tree species, density, basal area (BA), and heights, with ground litter, branches, and logs. Forest stand plots were centered on the logs in each block, and measured 20 × 25 m. Measurements were recorded pre-treatment (2016 to 2017) and annually during the post-treatment summers of 2018 to 2022.

We measured herbaceous vegetation and forest floor microhabitat variables (plant basal and canopy cover, moss, lichen, litter, bare soil, rock, and wood) annually in each sampling block on 3 replicated 55-m line-point intercept transects (Suazo et al. 2013). Line-point intercept data record occurrence frequency, from which we inferred percentage cover from the summarized point counts. Understory plant diversity (Shannon–Wiener index, *H′*; Shannon and Weaver 1949), species richness, and evenness (*J′*) were calculated from these data.

### Statistical Analyses of the Beetle Community and Populations

In this study, raw data were counts of each beetle species’ specimens collected per trap per month. We also used the term “abundance” as equivalent to “activity-density” (*sensu*  Halsall and Wratten 1988), recognizing that the number of specimens collected in passive-capture pitfall traps was a combined probability-of-capture function of each species’ absolute density coupled with its movement activity level.

Raw collection counts of beetles per trap per month were adjusted for small differences in collection periods each month, as collection dates each year did not fall exactly on the first day of the month (due to weather conditions, fire restrictions, and crew work schedules). Count data from each collection were divided by the actual number of days of the sample period, and then multiplied by 30.5 days to standardize monthly collections for June, July, August, and September. These values were then used in subsequent analyses.

The BACI design is a special case of a 2 group, parallel design with repeated measures. Therefore, we analyzed changes in beetle species abundances using generalized linear mixed models (GLMMs) in the SAS v9.4 statistical package (SAS Institute, Inc., Cary, NC, USA). For analyses presented here, we pre-averaged standardized abundance values over months within each year for each pitfall trap to obtain the abundance dependent variable, mean number/trap-year. Diversity index values were also averaged over months to obtain mean values for each trap-year. The analyses accommodated the nested split-plot design using fixed effects for treatment, year, log vs. non-log, and all 2- and 3-way interactions among main effects. Total variance was partitioned into 3 hierarchical levels that accounted for non-independence within and between levels: residual variation within traps across years (level 1), variation between log/non-log trap pairs (level 2, sub-plot variation), and variation between-log variation within treatment/control sites (level 3, whole-plot variation). In the standard hierarchical linear model framework, random effects were assumed to be independent and normally distributed, and although random effects are independent across levels, they capture the shared variation within levels. It is possible to use more complex variance components structures, such as temporally autoregressive correlation or spatially dependent correlation across units, but these models increase the number of parameters to estimate, and we found that they did not reliably improve model fit as evaluated by Akaike Information Criterion (AIC) or Bayesian Information Criterion (BIC).

The Kenward–Roger (1997) method was used to compute denominator degrees of freedom for *F*-tests and linear contrasts. Least squares adjusted means (LSM, ± standard errors) were used to summarize predicted group means, and custom linear contrasts between LSM were constructed to provide additional information about treatment × year interactions. The contrasts tested whether change from the previous year was different for control and treatment sites. *P*-values from the 5 change contrasts (2018–2017, 2019–2018, 2020–2019, 2021–2020, 2022–2021) were adjusted for multiple comparisons using the adaptive-Holm method (Hochberg and Benjamini 1990). The adaptive-Holm method was also used for other ad hoc multiple comparisons. Graphical methods were used to assess model assumptions, and variable transformations were applied if necessary to improve adherence to assumptions about normality and homogeneous variances. For this design, we detected statistically significant treatment × year interactions and the treatment × year × log interactions when these effects explained 3.5% of total variation, *η*^2^.

For beetle community diversity analyses, we utilized the Shannon–Wiener (*Hʹ*) diversity index with the associated values of species richness (*S*) and evenness (*Jʹ*) for each trap-year. We calculated these indices both for total annual values (all samples pooled) and for mean annual values per trap-year. As recommended by Roswell et al. (2021), we evaluated sampling coverage for rare species by year and treatment using the methods of Chao and Jost (2012). We also estimated sample completeness with species rarefaction curves constructed using the Chao-1 index (appropriate for count data in observed species abundances, *S_obs_*) to calculate the estimated total number of species (*S_est_*) in the treatment and control areas (Chao 1984); sample completeness was calculated as *S_obs_*/*S_est_*. We analyzed average annual community diversity measures using the GLMM approach described above for testing main effects of treatments, years, log/non-log trap locations, and up to 3-way interactions.

We also analyzed forest restoration treatment effects on the subset of species that were classified as saproxylic beetles (requiring dead or dying trees, logs, and other coarse woody debris as habitat for either adults or larval development). These species were generally either fungivores under bark or in rotting wood, or predators on other tree/log-dwelling insects (such as bark beetles). Saproxylic species were analyzed with the GLMM approaches described above, both for mean number of species per trap per year and the mean number of individuals collected per trap per year.

We conducted beetle community similarity analyses through time between treated and control sites using (1) Jaccard’s (1908) species similarity index, (2) non-metric multi-dimensional scaling (NMMDS) analyses, and (3) permutational multi-variate analysis of variance (PERMANOVA) software (PRIMER-E [Version 7], Albany, Auckland, New Zealand). In the NMMDS and PERMANOVA analyses, we used a *Log_(x + 1)_* data transformation and the Bray–Curtis similarity index with 999 permutations, and we evaluated the NMMDS results at the 50% and 75% similarity levels. The PERMANOVA analyzed similarity differences between treatments and among years with treatment × year interaction terms.

We analyzed the trophic structure of the beetle community to assess whether the treatments resulted in shifts through time in the proportions of species and numbers of individuals in different trophic groups. We classified each beetle species into a trophic category (predator, herbivore, omnivore, fungivore, and detritivore) using natural history literature resources (Johnson and Cameron 1969, Arnett and Thomas 2001, Arnett et al. 2002, Larochelle and Larivière 2003, and references therein). We tested the differences in the distributions of species and numbers of individuals across trophic groups for pairwise differences between treatments within years, and between years within treatments, using the 2-sample Kolmogorov–Smirnov (KS) test (Massey 1951).

We also assessed the association between species-specific beetle abundances and microhabitat variables (plant canopy and basal cover separated as grass, forb, sedge, sub-shrub, and vine, and forest floor variables of litter, bare soil, moss, rock, and wood). We undertook analyses for those “common” beetle species that showed significant abundance changes on the treatment site during the post-treatment years 2018 to 2022 and had sample sizes ≥ 20 individuals. We first performed a model selection procedure to identify significant model variables (Burnham and Anderson 2002). We identified models with ΔAIC_c_ <2.00, and with model evidence ratios of <3.00 (*w_i_*/*w_j_*, where *w_i_* is the model weight of the top model, and *w_j_* is the model weight of other models). We also computed Mallows *C_p_* statistic for co-linearity, in which *C_p_* values less than or equal to the number of parameters support model selection (Mallows 1973, Gilmour 1996). Once the “best” model was selected, we computed the model coefficients using multiple regression analyses. *P*-values reported were for 2-tailed probabilities. For these and the trophic group analyses, we used the software package Statistix 10 (Analytical Software, Tallahassee, FL, USA).

## Results

### Meteorological Dynamics

During the 6 years of our study, summer rainfall (June to August) ranged from 70 to 278 mm, while winter moisture (December to February) varied from 77 to 502 mm (Appendix A, Fig. S2). Air temperatures ranged from a low of –24.2 °C to 32.2 °C, and soil temperatures (at 2.5 cm depth) ranged from –13.5 °C to 44.6 °C. Snowpack depth varied over 6-fold, from 178 mm to 1,168 mm. The thinning treatment caused an increase in summer solar radiation from a daily mean of 76.1 µmol/m^2^s in 2017 to 416.4 µmol/m^2^s in 2018 (547% increase). Forest thinning also increased mean air temperatures by 0.7 °C, concomitant with a 10% reduction in mean relative humidity. Wind speeds also increased in the thinned area by 0.5 m/s (mean) and 2.0 m/s (maximum); see Parmenter and Losleben (2023) for additional details.

### Vegetation and Forest Floor Habitats

Detailed vegetation results are presented in Parmenter et al. (2026), and we briefly summarize them here and in the Supplemental Materials (Appendix A, Fig. S3, Table S1). The plant assemblage on the study site comprised 115 vascular plant species from 29 families, plus 7 species of (nonvascular) bryophytes (see Stout and Hall 2023 for species list and data). Forbs included 60 species, with 26 grass species, 11 shrubs and subshrubs, 9 sedges and rushes, 6 species of trees, 7 mosses, and 2 vines. The assemblage was dominated by 105 perennial species, with 17 annual or biennial species. Native species comprised 106 of the 122 total species, and 16 species were non-natives.

The thinning treatment reduced forest canopy cover from 72% to 40%, and the broadcast-slash burn and subsequent windthrow further reduced tree canopy cover to 38%. Thinning reduced live tree densities from 1,040/ha to 116/ha, and BA was reduced from 39 m^2^/ha to 7 m^2^/ha; the burn and additional windthrow reduced live tree density to 68/ha with a mean BA of 5 m^2^/ha.

On the forest floor, total herbaceous, shrub, and vine vegetation canopy cover declined from 62% to 30% after the ­thinning operations in 2018, but increased to 96% by 2022 (Appendix A, Fig. S3, Table S1). Temporal patterns of basal plant cover in the treatment area were similar to canopy cover dynamics. Plant community diversity and evenness showed no treatment effect but exhibited significant interannual dynamics (Appendix A, Fig. S3, Table S1). Analysis of plant species richness produced a significant treatment difference because the treatment area had more plant species than the control prior to treatment but showed no significant treatment × year interaction (Appendix A, Fig. S3, Table S1).

The forest floor variables of bare soil and moss exhibited significant treatment and year × treatment interaction effects (bare soil increased and moss decreased; Appendix A, Fig. S3, Table S1). Litter cover averaged 75% and was indistinguishable between treatment and control (Appendix A, Fig. S3, Table S1); however, during thinning, forest floor litter depth was reduced from 11.6 cm to 6.5 cm in the treatment, and the prescribed fire further reduced mean litter depth to 0.9 cm on the treatment compared to the control (mean depth of 9.4 cm). Rock cover had no treatment effect, while wood cover (logs, stumps, and branches) increased above control values during thinning but then declined below controls after the burn (Appendix A, Fig. S3, Table S1).

### Beetle Community Summary

During the study, we collected 55,761 beetle specimens from 289 taxa and 51 families (Appendix A, Table S2). Based on species numbers, the collected samples were dominated by Staphylinidae (45 taxa) and Carabidae (40 taxa), followed by Leiodidae (21), Curculionidae (20), Cerambycidae (13), Cryptophagidae (12), Elateridae (12), Chrysomelidae (11), Nitidulidae (11), and Coccinellidae (10). In terms of specimen counts, the dominant families were Carabidae (32,995 specimens), Staphylinidae (11,098), Cryptophagidae (5,137), Latridiidae (1,949), and Tenebrionidae (1,192). A total of 80 species (28%) were represented by only a single specimen. While we acknowledge that pitfall traps may be neither effective nor efficient in collecting rarer beetle taxa, these rare species are nonetheless a component (albeit potentially ephemeral) of the surface-active beetle fauna, and we have included them in our community analyses.

### Beetle Community Diversity

**Fig. 4.**
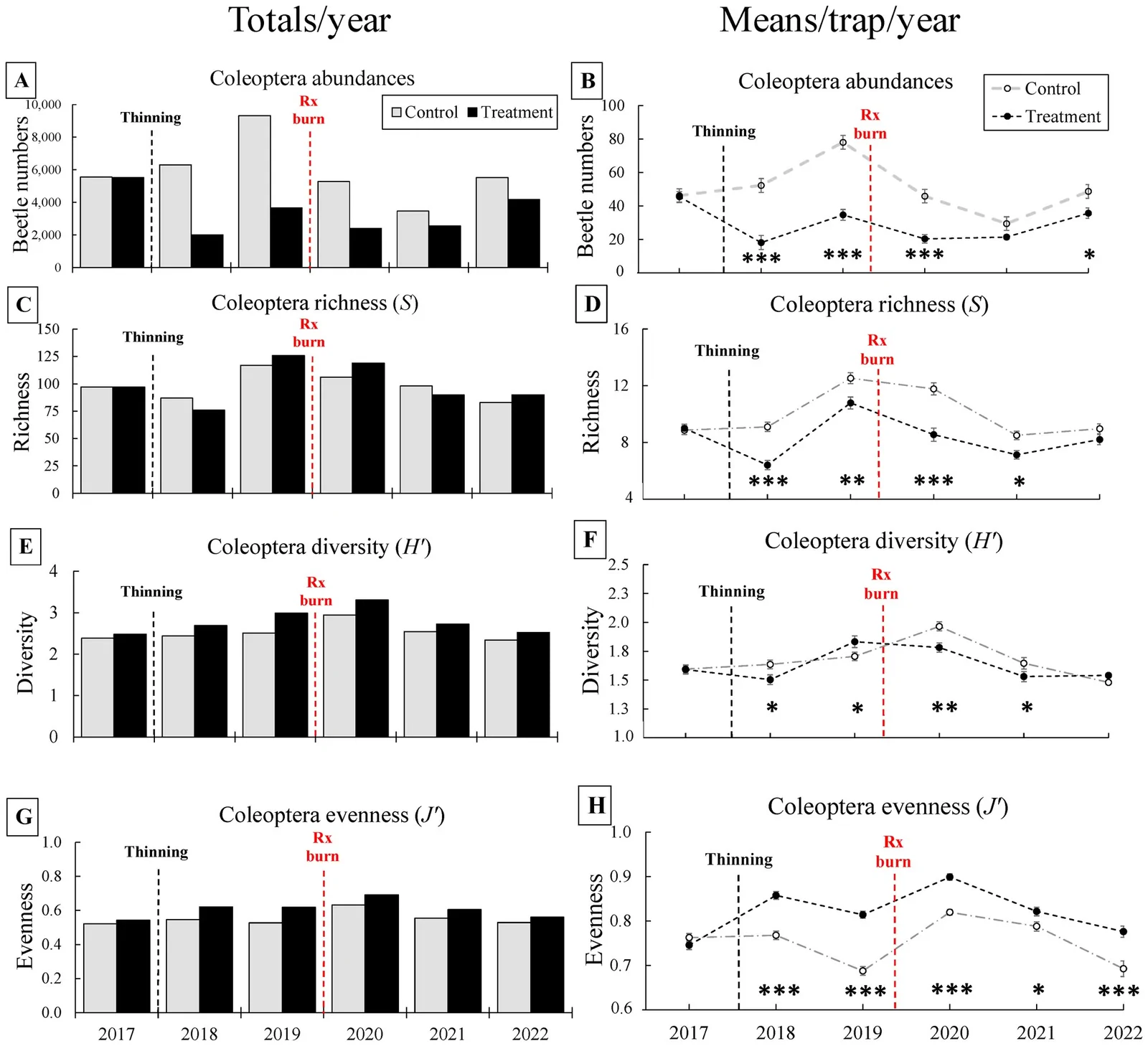
Results of forest restoration treatment effects through time on surface-active beetle abundances (A, B), species richness, *S* (C, D), diversity, *Hʹ* (E, F), and evenness indices, *Jʹ* (G, H). Bar charts show annual totals, and line graphs with data points show means of annual trap collection values during each year ± SE. Asterisks indicate significant treatment differences within years (see Appendix A, Table S3 for GLMM results): * = *P *≤ 0.05; ** = *P *≤ 0.01; *** = *P *≤ 0.001.

We observed strong statistical support from the GLMM analyses (*P *< 0.001) for changes in total beetle abundances, species richness, diversity, and evenness (per trap per year) through years and a treatment × year interaction (Fig. 4, Appendix A, Table S3), although patterns diverged somewhat between total annual values versus sample means/trap-year. All measures were nearly identical during the 2017 pre-treatment sampling period (Fig. 4). Total beetle abundances declined following treatment for both annual totals (Fig. 4A) and mean values per trap (Fig. 4B). Species richness values produced different patterns for annual totals versus mean richness per trap-year, in that total richness in the treatment area remained close to control values throughout the study (Fig. 4C), but for mean richness/trap-year, values significantly decreased in the treatment sites following thinning and burning before becoming equal to the controls in 2022 (Fig. 4D). Evaluation of species richness sampling completeness using Chao-1 rarefaction curves indicated that even with 30 traps and continuous sampling, we still missed a number of rare species, with completeness values ranging from 0.55 to 0.79 (Table 1, Appendix A, Fig. S4). Over the 6 years of our study, the Chao-1 estimate indicated that the sampling in the Control area underestimated rare species richness by a mean of 38.3 per year, while the treatment area was underestimated by 48.6 species per year (Table 1); these mean values were not significantly different (paired 2-tailed *t*-test, *t *= 1.03, *df *= 5, *P *= 0.350). However, based on the large numbers of individual beetles collected, our sample richness coverage values based on the Chao and Jost (2012) index were very high, ranging from 0.984 to 0.997 (Table 1).

**Table 1.** Annual sample completeness (Chao-1 index) and sample coverage values for Coleoptera species richness (*S*) on control and treatment sites in the Cerro Seco Unit 5 project area, Valles Caldera National Preserve, NM

Sample completeness				
Year	Treatment	No. spp. Observed *S_obs_*	No. spp. Estimated *S_est_*	Completeness (*S_obs_/S_est_*)
**2017**	Control	97	127.0	0.76
	Pre-treatment	97	133.3	0.73
**2018**	Control	87	136.0	0.64
	1-year post-thin	76	126.0	0.60
**2019**	Control	117	156.4	0.75
	2-year post-thin	126	180.3	0.70
**2020**	Control	106	136.7	0.78
	1-year post-burn	119	163.7	0.73
**2021**	Control	98	156.9	0.62
	2-year post-burn	90	124.0	0.73
**2022**	Control	83	105.0	0.79
	3-year post-burn	90	162.2	0.55

Sample coverage					
Year	Treatment	*n*	*f_1_*	*f_2_*	Coverage
**2017**	Control	5,545	30	15	0.995
	Pre-treatment	5,522	33	15	0.994
**2018**	Control	6,302	28	8	0.996
	1-year post-thin	2,009	30	9	0.985
**2019**	Control	9,320	32	13	0.997
	2-year post-thin	3,666	39	14	0.989
**2020**	Control	5,270	26	11	0.995
	1-year post-burn	2,400	39	17	0.984
**2021**	Control	3,467	36	11	0.990
	2-year post-burn	2,554	33	16	0.987
**2022**	Control	5,524	21	10	0.996
	3-year post-burn	4,182	38	10	0.995

See Appendix A, Fig. S4 for Chao-1 rarefaction curves.

*n* = number of individuals collected.

*f_1_* = number of species with only 1 individual.

*f_2_* = number of species with only 2 individuals.

Coverage = values based on Chao and Jost (2012).

Diversity indices for annual totals exhibited a small, consistent increase in post-treatment years (Fig. 4E), but on a mean/trap-year basis, diversity initially declined on the treatment sites relative to the controls in 2018 after thinning, before increasing above controls in 2019 (Fig. 4F). Diversity on the treatments declined again following the burn in 2020, before equilibrating with the control in 2021 and 2022 (Fig. 4F).

Evenness indices for both annual totals and means/trap-year were statistically equal in 2017 but increased in the treatment sites relative to controls following thinning and burning, remaining higher for the duration of the study (Fig. 4G and H, Appendix A, Table S3). Delving into these results, we parsed the number of collected specimens and abundance percentages of total beetles collected each year into the lower, middle, and top thirds of ranked-order species abundances (following protocols in Crowder et al. 2012) and found that the higher evenness values following forest thinning and burning treatments resulted from the reduction in specimens ­collected from common species (eg *Carabus taedatus* Fabricius, *Pterostichus adstrictus* Eschscholtz, *P. restrictus* Casey [Carabidae], *Cryptophagus tuberculosus* Mäklin [Cryptophagidae], and Aleocharinae spp. [Staphylinidae]) coupled with only minor changes in abundances of rarer species; this pattern increased the percentage abundances of the rarer species in the lower and middle thirds of ranked-order species (see Appendix A, Fig. S5).

### Beetle Community Trophic Structure

**Fig. 5.**
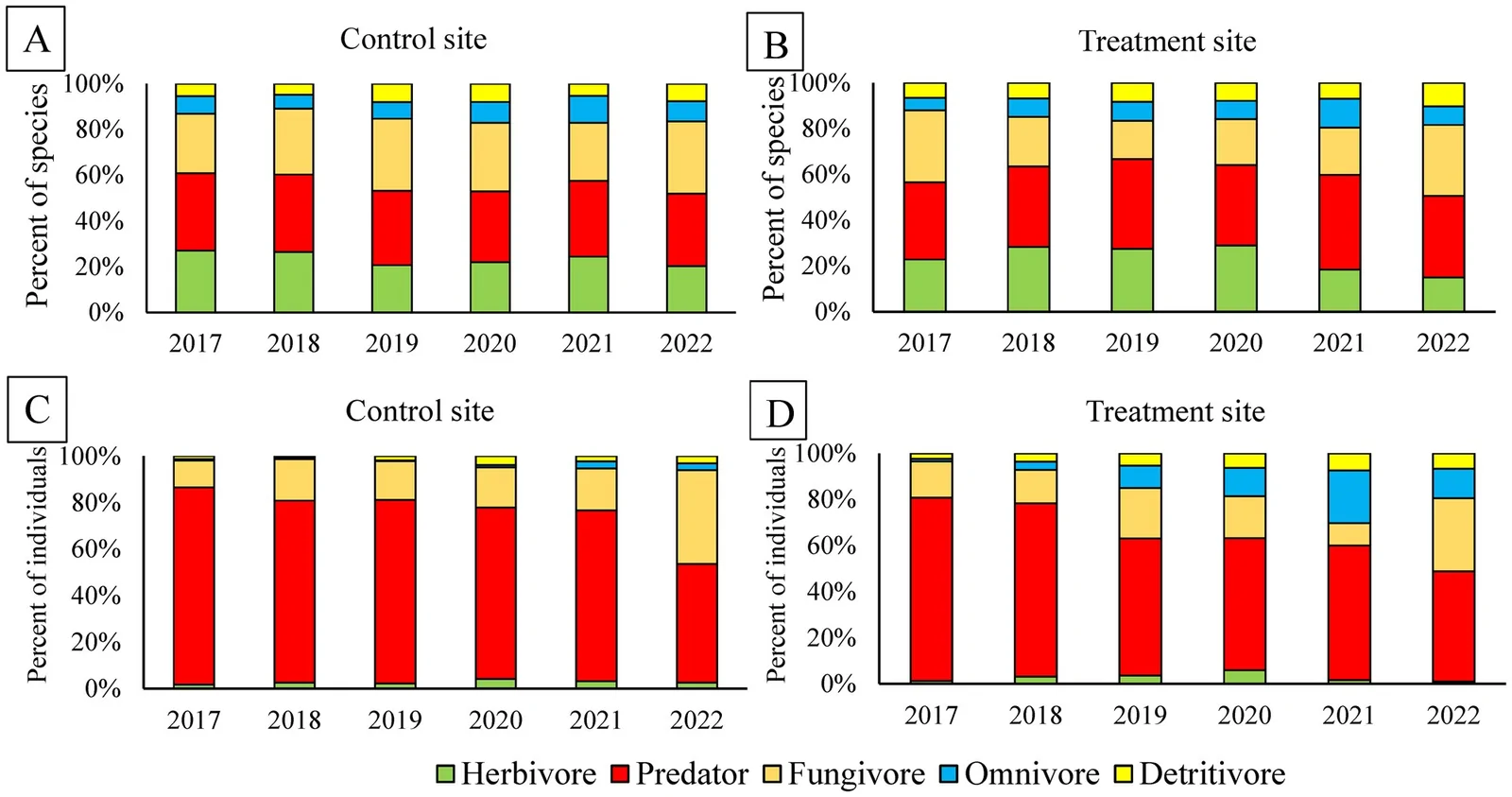
Proportions of surface-active beetle trophic groups through time in control and treatment sites. (A, B) Species numbers. (C, D) Numbers of individuals. See Appendix A, Table S4 for Kolgomorov–Smirnov test results.

We found that the adult beetle community was composed of 79 taxa of predators, 83 herbivores, 18 omnivores, 82 fungivores, and 18 detritivores; 9 taxa lacked published information on trophic status (Appendix A, Table S2). Our analyses indicated that the species composition of the beetle community trophic structure did not change through time or shift as a result of the forest treatments (thinning or fire), though there was some evidence that the numbers of individuals comprising the trophic groups shifted on the treatment sites following forest thinning and burning. Results of the *KS* tests produced no significant differences in distributions of trophic groups’ species proportions between years or between treatments and controls within years (Fig. 5; *KS* values ranged from 0.04 to 0.15, *P *= 1.00 to 0.17; Appendix A, Table S4), while significant differences in the numbers of individuals in trophic groups were observed in the treatment sites between 2017 and post-treatment years (Fig. 5; *KS* values ranged from 0.04 to 0.32, *P *= 1.00 to 0.00; Appendix A, Table S4). These shifts included increased numbers in the treatment areas of omnivores, fungivores, and detritivores beginning in 2019 (Fig. 5).

### Saproxylic Beetle Assemblage

**Fig. 6.**
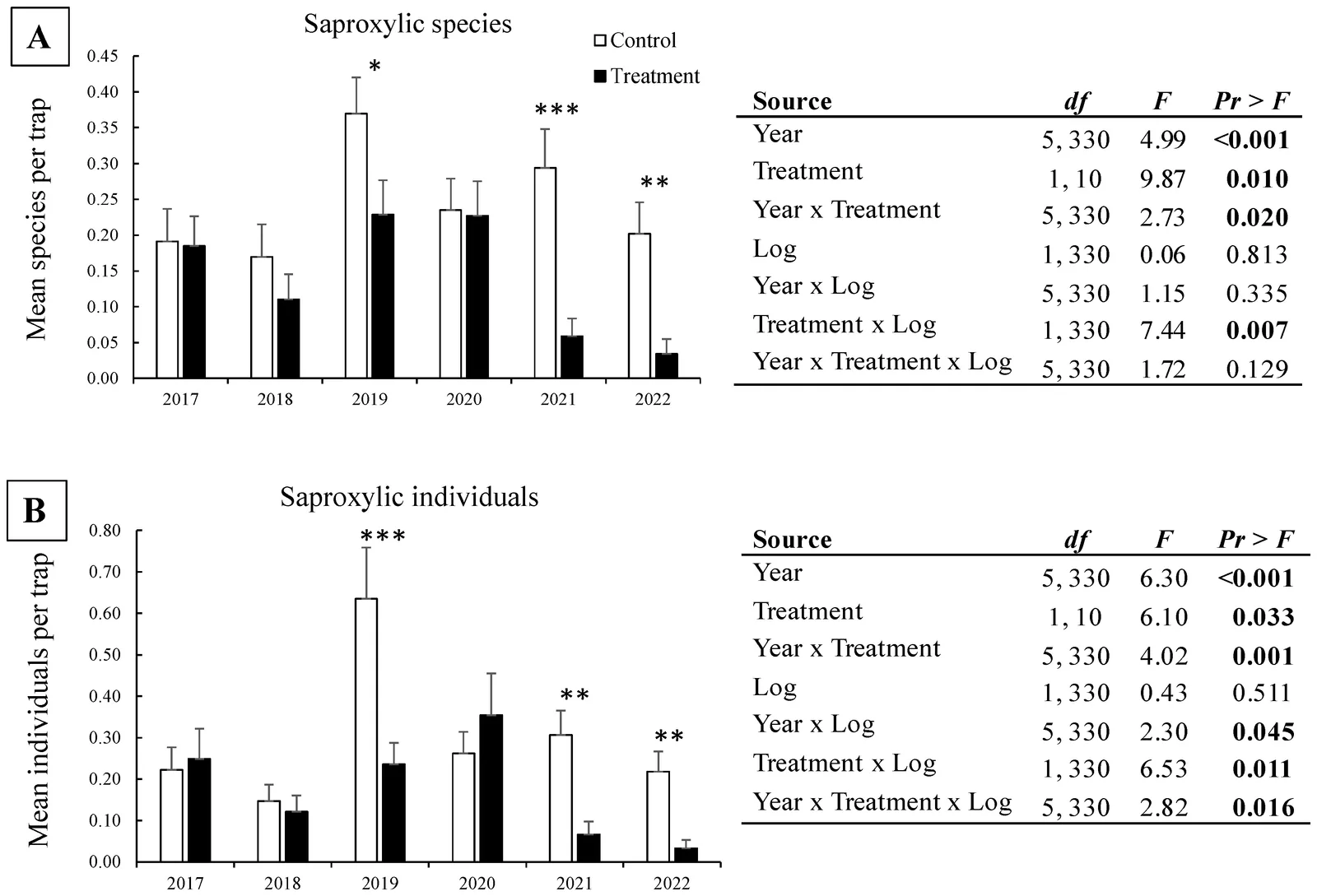
Saproxylic beetle species through time. Panels A and B display means per trap per year ± SE. GLMM analysis results are shown in tables at right. Asterisks indicate significant differences between treatments within years based on custom linear contrasts between least squared adjusted means (see text): * = *P *≤ 0.05; ** = *P *≤ 0.01; *** = *P *≤ 0.001.

A total of 48 beetle species (17%) were saproxylic (Appendix A, Table S2). We found that both the mean number of saproxylic beetle species and the number of individuals collected per trap per year were smaller in the post-thinning treatment sites in 2019 relative to controls, and then further declined 2 and 3 years after the broadcast slash burn in autumn 2019, with significant overall treatment effects, year effects, and treatment × year interactions (Fig. 6). In addition, the presence of logs had no consistent effect on either saproxylic species or their abundances (see statistical results in Fig. 6), although abundances were higher in non-log sites in controls in 2019 and then higher in treatment log sites in 2020 and 2021 (as indicated in significant log × treatment × year interaction terms in Fig. 6).

### Beetle Community Similarities

Using Jaccard’s (1908) species similarity indices to compare control and treatment beetle communities, we found that the 2017 pre-treatment sites shared 51.6% of their resident species, but this similarity dropped through time following treatments to 35.1% by 2022 (Table 2). Variability in species similarity between years within the control site during the study ranged from 42.9% to 55.0%. In contrast, between-year species similarity in the treatment site produced lower values, ranging from 28.1% to 43.0% (Table 2), indicating greater divergence of the beetle community following treatments.

**Table 2.** Number of Coleoptera species and Jaccard’s species similarity index comparisons by treatments and years, Cerro Seco Unit 5 study site, Valles Caldera National Preserve, NM

Year	Total no. species	Control no. species	Treatment no. species	No. common species	No. unique species in Control	No. unique species in Treatment	Jaccard’s similarity index
**Comparisons between treatment and control**							
**2017**	128	97	97	66	31	31	0.516
**2018**	112	87	76	51	36	25	0.455
**2019**	171	118	127	74	44	53	0.433
**2020**	155	106	119	70	36	49	0.452
**2021**	138	98	90	50	48	40	0.362
**2022**	128	83	90	45	38	45	0.351
**Comparisons between years: control site**							
**Years**	Total no. species	2017 no. species	Yr X no. species	No. common species	No. unique species in 2017	No. unique species in Yr X	Jaccard’s similarity index
**2017–2018**	121	97	87	63	34	24	0.521
**2017–2019**	149	97	118	66	31	52	0.443
**2017–2020**	131	97	106	72	25	34	0.550
**2017–2021**	131	97	98	64	33	34	0.489
**2017–2022**	126	97	83	54	43	29	0.429
**Comparisons between years: treatment site**							
**Years**	Total no. species	2017 no. species	Yr X No. species	No. common species	No. unique species in 2017	No. unique species in Yr X	Jaccard’s similarity index
**2017–2018**	122	97	76	51	46	25	0.418
**2017–2019**	165	97	127	59	38	68	0.358
**2017–2020**	151	97	119	65	32	54	0.430
**2017–2021**	146	97	90	41	56	49	0.281
**2017–2022**	143	97	90	44	53	46	0.308

**Fig. 7.**
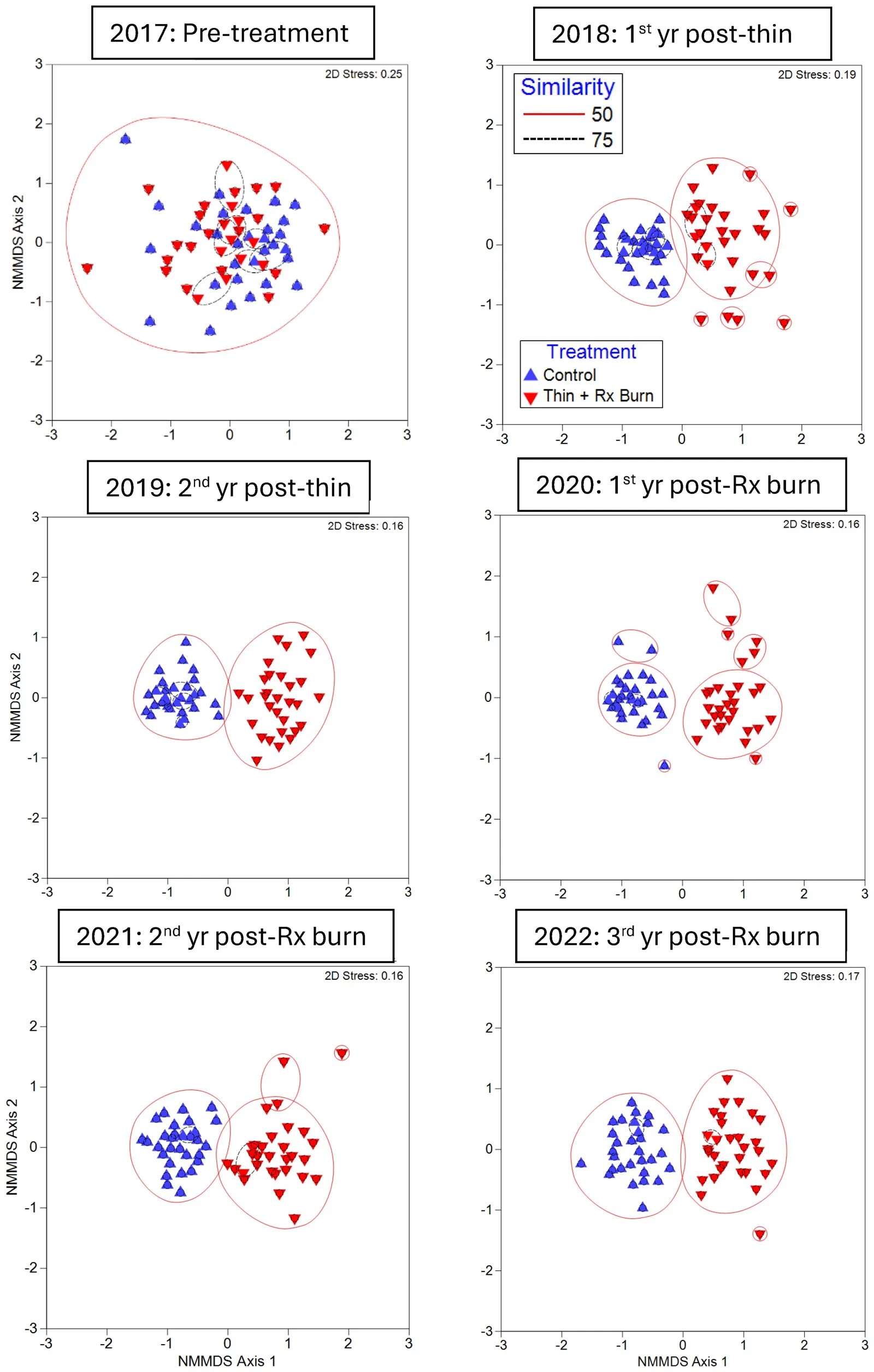
Non-metric multi-dimensional scaling (NMMDS) graphs of surface-active beetle communities during forest restoration treatments (2017 to 2022) on the Cerro Seco Unit 5 project site. 2017: Pre-treatment. 2018: first-year post-thinning. 2019: second-year post-thinning. 2020: first-year post-burn. 2021: second-year post-burn; 2022: third-year post-burn.

Applying the Bray–Curtis similarity index in the NMMDS analysis, we observed that in the pre-treatment (2017) period, the trap samples clustered together with >50% similarity, but following the thinning in winter 2017–2018, the 2018 treatment samples had separated from the controls (Fig. 7). The beetle communities remained distinct throughout the remainder of the post-treatment study period (2018 to 2022). The PERMANOVA analysis supported the Jaccard’s and Bray–Curtis NMMDS results, indicating highly significant effects of treatments, years, and treatment × year interactions (Table 3).

**Table 3.** Results of PERMANOVA analyses of annual Coleoptera Bray-Curtis similarities between treatments (control vs. thinned-burned) and among years (2017 to 2022) on the Cerro Seco Unit 5 project site, Valles Caldera National Preserve, NM

Source	*df*	SS	MS	Pseudo-*F*	*P* (perm)	No. of unique permutations
**Treatment**	1	101,570	101,570	84.952	0.001	999
**Year**	5	122,120	24,424	20.429	0.001	999
**Treatment × year**	5	64,835	12,967	10.846	0.001	999
**Residuals**	348	416,070	1,196			
**Total**	359	704,590				

### Beetle Population Dynamics

**Fig. 8.**
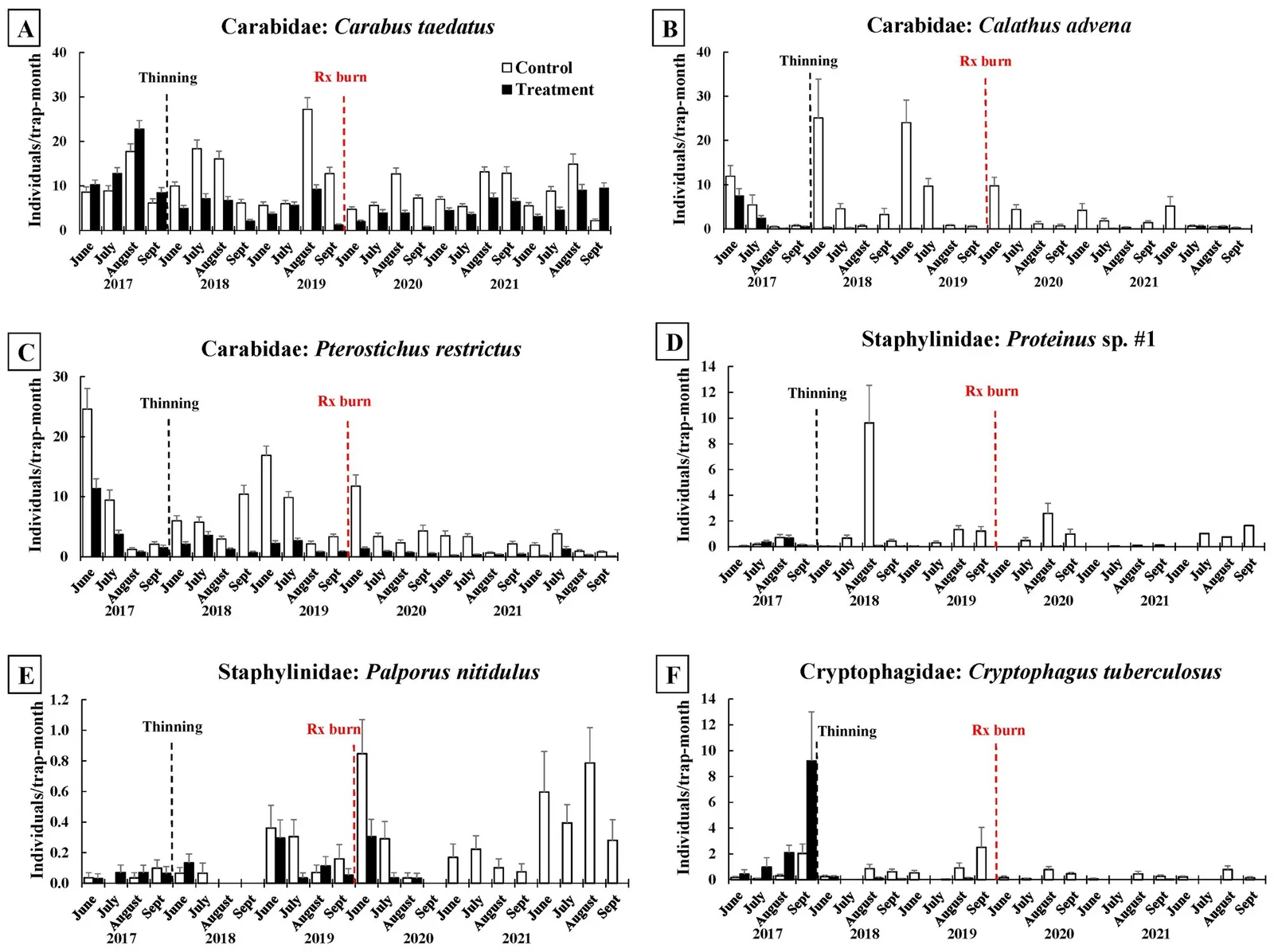
Examples of abundances of beetle species that decreased through time after forest restoration treatments on the Cerro Seco Unit 5 project area. Graphs display mean numbers of individuals per trap per month ± SE. See Appendix A, Table S5, for GLMM results.

**Fig. 9.**
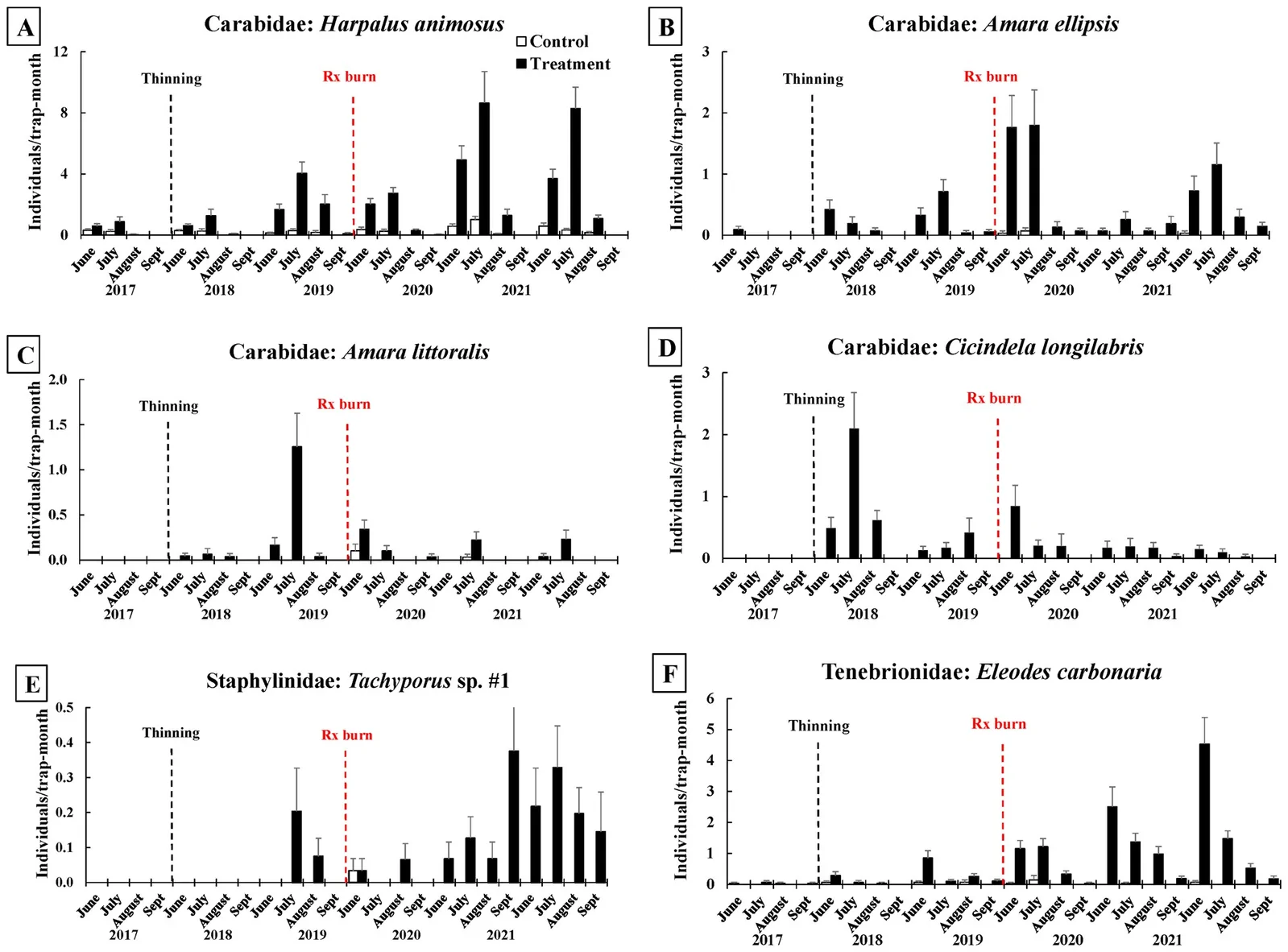
Examples of abundances of beetle species that increased through time after forest restoration treatments on the Cerro Seco Unit 5 project area. Graphs display mean numbers of individuals per trap per month ± SE. See Appendix A, Table S5, for GLMM results.

From the species-specific GLMM analyses for population abundances, 82 species met our criterion for ≥20 individuals in the samples. We found significant forest treatment-induced changes in abundance of 56 beetle species, with 21 species exhibiting population increases as a result of thinning and burning, and 35 species showing population decreases; 26 species showed no treatment response (Table 4; Appendix A, Table S5). Of the 35 common species that declined following treatments, many persisted in the treatment areas at lower abundances, though 3 species appeared to be extirpated: *Proteinus* sp. #1 (Staphylinidae) was not collected after 2018 (post-thinning), and *C. tuberculosus* (Cryptophagidae) disappeared after the burn in 2019; *Palporus nitidulus* (Fabricius) (Staphylinidae) also was not collected after 1-year post-burn in 2021 to 2022 (see examples in Fig. 8). Similarly, of the 21 common species in the post-treatment traps that had become more abundant following thinning and burning, 2 species (Carabidae: *Cicindela longilabris* Say; Staphylinidae: *Philonthus* sp. #1) were new colonists to the post-treatment site and were never collected in the control forest (see examples in Fig. 9).

**Table 4.** Responses of beetle species abundances to forest treatments on the Cerro Seco study site, Valles Caldera National Preserve, NM

Species increasing in abundance after treatment	Species decreasing in abundance after treatment	Species showing no response to treatment
Carabidae	Carabidae	Carabidae
*Agonum placidum*	*Calathus advena*	*Cymindus cribricollis*
*Amara ellipsis*	*Calathus ingratus*	*Notiophilus semistriatus*
*Amara littoralis*	*Carabus taedatus*	*Scaphinotus snowi*
*Cicindela longilabris*	*Notiophilus directus*	
*Harpalus animosus*	*Pterostichus adstrictus*	Chrysomelidae
*Harpalus ellipsis*	*Pterostichus restrictus*	*Phyllotreta* sp. #1
*Harpalus nigritarsis*		
*Harpalus somnulentus*	Cryptophagidae	Cryptophagidae
*Rhadine nivalis*	*Atomaria* sp. #2	*Atomaria* sp. #1
*Sericoda bembidioides*	*Cryptophagus pilosus*	
*Sericoda quadripunctata*	*Cryptophagus tuberculosus*	Curculionidae
*Synuchus dubius*		*Otiorhynchus ovatus*
	Curculionidae	
Coccinellidae	*Thricolepis inornata*	Elateridae
*Hippodamia convergens*		*Athous arizonicus*
*Hippodamia parenthesis*	Elateridae	*Athous* sp. #2
	*Limonius aeger*	*Stropenron pudica*
Curculionidae	*Stropenron* sp. #1	
*Hylastes gracilis*		Endomychidae
*Pseudorimus orbicollis*	Latridiidae	*Aphorista morosa*
	*Corticaria* sp. #1	
Silphidae	*Latridius* sp. #1	Histeridae
*Heterosilpha ramosa*		*Hister furtivus*
	Nitidulidae	*Saprinus* sp. #1
Staphylinidae	*Epuraea alternans*	
*Philonthus* sp. #1		Latridiidae
*Tachyporus* sp. #1	Ptiliidae	*Corticarina* sp. #1
	*Acrotrichis* sp. #1	
Tenebrionidae		Leiodidae
*Eleodes carbonaria*	Scarabaeidae	*Agathidium pulchrum*
	*Dichelonyx* sp. #1	*Catops* sp. #1
Trachypachidae		*Hydnobius longidens*
*Trachypachus inermis*	Scraptiidae	*Ptomaphagus* sp. #1
	*Anaspis rufa*	
		Silphidae
	Staphylinidae	*Nicrophorus investigator*
	*Acrolocha* sp. #1	
	*Acrotona* sp. #1	Staphylinidae
	*Aleochara* sp. #1	*Belonuchus* sp. #2
	Aleocharinae spp.	*Brachycepsis* sp. #1
	*Anotylus* sp. #1	*Carpelimus* sp. #1
	*Bobitobus* sp. #1	*Gabrius* sp. #1
	*Bolitobius* sp. #1	*Myrmecocephalus arizonicus*
	*Lordithon* sp. #1	*Oxytelus* sp. #1
	*Megarthrus angulicollis*	*Stenus* sp. #1
	*Palporus nitidulus*	
	*Phyllodrepa* sp. #1	Tenebrionidae
	*Phyllodrepa* sp. #2	*Eleodes snowii*
	*Proteinus* sp. #1	
	*Pseudopsis* spp.	
	*Quedius* sp. #1	
	*Xenota* sp. #1	
	Tetratomidae	
	*Tetratoma concolor*	

Species listed had >20 individuals collected; see Appendix A, Table S5 for GLMM statistical results.

### Beetle Associations With Microhabitat Variables

From the GLMM results, we found that logs had a significant effect on the distributions of 23 beetle species (Table 5). Of these 23 species, 8 species were collected more often next to logs, while 15 species were collected more often away from logs. Only a single species (*Sericoda bembidioides* Kirby; Carabidae) was collected exclusively next to logs; the other 22 of 23 species were found in both log and non-log microsites (Table 5).

**Table 5.** Results of beetle species’ associations with significant log versus non-log microhabitat differences on the Cerro Seco study site in Valles Caldera National Preserve, NM

Family/species	Log sites	Non-log sites	*P*-value
Carabidae			
*Amara ellipsis*	0.103 ± 0.018	**0.265 ± 0.042**	0.002
*Harpalus animosus*	0.650 ± 0.062	**1.434 ± 0.147**	<0.001
*Sericoda bembidioides*	**0.137 ± 0.049**	0.000 ± 0.000	0.007
*Sericoda quadripunctata*	**0.233 ± 0.039**	0.089 ± 0.020	<0.001
Coccinellidae			
*Hippodamia convergens*	0.032 ± 0.011	**0.110 ± 0.031**	0.044
Cryptophagidae			
*Atomaria* sp. #1	1.427 ± 0.208	**2.528 ± 0.420**	0.031
*Cryptophagus pilosus*	0.910 ± 0.088	**1.121 **± 0.094	0.048
Elateridae			
*Athous* sp. #2	0.036 ± 0.008	**0.670 ± 0.013**	0.031
*Stropenron* sp. #1	0.087 ± 0.022	**0.203 ± 0.032**	<0.001
Endomychidae			
*Aphorista morosa*	**0.053 ± 0.014**	0.021 ± 0.006	0.035
Histeridae			
***Hister furtivus***	0.025 ± 0.008	**0.084 ± 0.020**	0.009
**Nitidulidae**			
*Epuraea alternans*	0.158 ± 0.026	**0.334 ± 0.044**	<0.001
Scraptiidae			
*Anaspis rufa*	0.047 ± 0.010	**0.119 ± 0.022**	0.002
Staphylinidae			
*Myrmecocephalus arizonicus*	**0.032 ± 0.007**	0.010 ± 0.004	0.013
*Philonthus* sp. #1	0.006 ± 0.003	**0.024 ± 0.007**	0.016
*Phyllodrepa* sp. #1	**0.098 ± 0.024**	0.023 ± 0.008	0.002
*Proteinus* sp. #1	**0.698 ± 0.125**	0.299 ± 0.087	0.005
*Pseudopsis* spp.	0.034 ± 0.008	**0.090 ± 0.016**	0.004
*Quedius* sp. #1	**0.244 ± 0.032**	0.117 ± 0.020	<0.001
*Stenus* sp. #1	0.016 ± 0.005	**0.032 ± 0.007**	0.045
*Tachyporus* sp. #1	0.026 ± 0.007	**0.055 ± 0.012**	0.034
Tenebrionidae			
*Eleodes carbonaria*	0.250 ± 0.033	**0.461 ± 0.058**	<0.001
*Eleodes snowii*	**0.585 ± 0.047**	0.384 ± 0.041	<0.001

Values are mean (±SE) number of individuals collected per trap per month during the study; only species with >20 individuals included in analyses (see Appendix A, Table S5 for GLMM statistical results). Boldface values show preferred microhabitat.

We conducted model selection and multiple regression analyses for associations between beetle abundances and microhabitat variables on 38 species with ≥ 20 individuals collected in the post-treatment traps during 2018–2022. A total of 21 species showed abundance increases (Appendix A, Table S6) and 17 species showed abundance decreases (Appendix A, Table S7). The importance of microhabitat variables varied considerably among the beetle species, with no consistent patterns of positive or negative relationships with beetle abundances for particular habitat variables (Appendix A, Tables S6 and S7). Adjusted-*R*^2^ values for the “best models” ranged from 0.052 to 0.906, with a mean adjusted-*R*^2^ (± SE) for increasing species of 0.495 ± 0.049 and 0.498 ± 0.039 for decreasing species.

## Discussion

### Forest Treatment Impacts

During our study, we documented the dynamics of 289 taxa of surface-active beetles and found that the forest restoration treatments distinctly changed the composition of the beetle community. Beetle abundances, richness, diversity, and evenness were comparable in treatment and control areas prior to treatment, but mean total abundance and species richness per trap-year declined in the treatment sites immediately following the tree thinning and again after the prescribed broadcast-slash burn, with eventual near-recovery in the following 3 years. Diversity also declined immediately following treatments, but recovered within 1 to 2 years later. Evenness indices increased and maintained higher values in the treated forest following thinning and burning.

In a meta-analysis of 93 published studies on the effects of forest prescribed fires on species diversity, abundances, richness, and evenness, Crowder et al. (2012) examined the components of diversity and found that fire treatments increased organism abundances and evenness, but not species richness. Crowder et al. (2012) attributed the increase in evenness to disproportionate abundance gains of species in the lower third of the rank-abundance continuum in contrast to the more abundant taxa. While our results showed post-treatment decreases in total beetle abundances and species richness, the beetle community evenness indices exhibited significant increases. These evenness dynamics were driven not by abundance increases in rarer species (as noted by Crowder et al. 2012), but rather by declines in abundances of common species which created the statistical effect of increasing the proportional contribution of rarer species and raising the evenness values.

As expected, a number of the common beetle taxa exhibited significant changes in abundances, with 21 taxa increasing and 35 taxa decreasing following the treatments. Three common species appeared to be extirpated from the treatment plots, while 2 new beetle species arrived as colonists. Of the new colonists, the predatory tiger beetle (*C. longilabris*; Carabidae) typically prefers open habitats with bare soil (Knisley et al. 2023), and quickly colonized the thinned forest, but declined in abundance through time as herbaceous vegetation developed (Fig. 9D). Similarly, the predatory *Philonthus* sp. #1 (Staphylinidae) colonized a year after thinning, but also declined as herbaceous plant ground cover increased.

The proportion of taxa in different trophic groups did not change following treatments, although the numbers of individuals in the omnivore, fungivore and detritivore groups increased in the post-treatment site. Saproxylic species and numbers of individuals generally declined as a result of the thinning operation, and then declined further 2 and 3 years after the slash broadcast burn. All of these changes led to reduced community similarities between the treatment and control forests, creating a distinctly different surface-active beetle community in the more open, grassy sub-canopy environment.

The results of our study are consistent with the few previous studies on thinning and burning operations conducted in coniferous forests in the western United States. Apigian et al. (2006a, b) compared leaf litter arthropod communities in mixed-conifer forests of the California Sierra Nevada range using a BACI design to test for differences in communities on sites treated with mechanical thinning + mastication, prescribed fire, thinning + mastication + fire, and untreated controls. For beetle communities (256 species in 49 families, 11,815 specimens), they found that community change from burning treatments was due to increases in rare species, as well as shifts in species-specific abundances of common species. Abundant families included Carabidae, Staphylinidae, Throscidae, and Cryptophagidae. These results compare well with our study, in which we also documented shifts in abundances of species, with less common open-habitat species becoming much more abundant in the more open, grass-dominated habitats of the post-treatment sites, and forest-habitat specialists declining in abundance; however, as noted above, most of our rare species did not show substantial increases in abundance.

In northern Arizona’s ponderosa pine forests, Villa-Castillo and Wagner (2002) monitored ground beetles (Carabidae, 20 species) for 3 years, comparing stands that had either been thinned, thinned and burned in prescribed fires, impacted by wildfires, or undisturbed. Carabid diversity and richness increased with disturbance level, with highest values for wildfire-impacted stands and stands that had been both thinned and burned. In our study, our overall beetle diversity and richness declined after treatments (Fig. 4), but when considering our Carabidae alone (40 species), our ground beetle species richness also increased following the prescribed fire from 18 species to 30 species, compared to the controls with an increase from 16 to 17 species.

Chen et al. (2006) examined both ground beetles (Carabidae, 22 species) and darkling beetles (Tenebrionidae, 27 species) in this same area of Arizona, and reported similar results to those of Villa-Castillo and Wagner (2002) for ground beetles, with higher diversity and richness in thinned and burned sites compared to undisturbed controls. Darkling beetles exhibited similar diversity and richness values among thinned and undisturbed stands (Chen et al. 2006). Our study site supported only 6 species of darkling beetles, only 2 of which occurred in the control forest sites (*Eleodes snowii* Blaisdell and *E. carbonaria* (Say)). *E. snowii* dominated the forest sites, and while *E. carbonaria* was less common in forested habitat, this latter species increased following treatment to become the numerically dominant darkling beetle in the more open post-treatment environment (Fig. 9).

### Beetle Community Trophic Structure

The trophic structure’s species composition of the beetle community did not change as a result of the forest treatments during our project (Fig. 5A and B; Appendix A, Table S4), although the numbers of individuals in the omnivore, fungivore and detritivore groups increased on the treatment sites at the expense of predators (Fig. 5D) compared to the pre-treatment (2017) community. Taxa contributing to these shifts in abundance included post-treatment increases in the carabid genus *Harpalus* (Fig. 9A), species of which are omnivorous and also feed on a variety of seeds, and a large increase in the tenebrionid darkling beetle, *Eleodes carbonaria* (Fig. 9F), which is a common detritivore as an adult in grasslands on the Preserve. Fungivores that increased post-treatment included the staphylinid rove beetle *Tachyporus* sp. #1 (Fig. 9E), the cryptophagid *Atomaria* sp. #1, along with several genera in Leiodidae and Endomychidae (Appendix A, Table S2). All these taxa appeared to be tracking changes in the habitat and food resources brought about by the forest treatments and vegetation successional responses (Appendix A, Tables S6 and S7).

Previous studies on forest disturbances and beetle trophic structure in the United States have produced mixed results. In Florida pine scrub, Greenberg and Thomas (1995) examined forestry practices (high-severity burning with salvage logging, clearcutting/chipping and clearcutting, and untreated controls) on the beetle community 5 to 7 years after treatments and found trophic guild structure was unchanged among treatments; these results were consistent with our findings. In contrast, Ferrenberg et al. (2006) assessed ground-dwelling arthropods (multiple classes and orders) in mixed-conifer forests in the California Sierra Nevada following prescribed fires in spring versus autumn compared to unburned controls. They found that fire reduced arthropod abundances but increased diversity compared to unburned mature forest and shifted arthropod trophic structure (burned areas had greater proportions of phytophagous, parasitic and xylophagous species, and a smaller proportion of detritivores); however, Coleoptera taxa were not separated in these analyses, and it was not clear if the beetle community’s trophic structure was affected by fire. On our study site in New Mexico, the beetles’ trophic structure composition did not show a significant change, but Parmenter et al. (2026) reported large increases in grasshoppers and crickets (Orthoptera) on our site following treatments, which clearly would inflate the herbivore/omnivore trophic group if other arthropod taxa were combined. Additional studies on forest restoration impacts on arthropod trophic structure are clearly needed.

### Saproxylic Beetle Assemblage

Forest restoration activities have substantial effects on the prevalence of snags, stumps, and coarse woody debris in treated areas, and this can impact the assemblage of saproxylic beetle species. The importance of logs on the forest floor has been recognized for over a century, as Shelford (1913) identified logs as a “stratum” and noted the succession of invertebrates through the decomposition process, and Adams (1915) detailed the groups of animals inhabiting decaying logs from initial tree death through the humus stage. Other early researchers expanded on these studies, describing insect succession processes in logs in various settings (eg Blackman and Stage 1918, 1924, Graham 1925, Savely 1939), a theme that has continued through the more recent literature (eg Grove 2002, Sandström et al. 2019, Thorn et al. 2020). Our study provides additional evidence in support of retaining logs and other coarse woody debris to sustain saproxylic species, given the decrease we observed in this group of beetles (both species and individuals) following the forest thinning and the subsequent low-intensity broadcast slash burn. We found 8 common species that were collected in significantly greater numbers next to logs (Table 5), although we could not distinguish whether the log was an important habitat resource versus simply acting as a “drift fence” to enhance trap captures (see Latty et al. 2006, Hanula et al. 2009). The slash burn eliminated much of the smaller-diameter wood from the forest floor (except stumps and some larger logs), which corresponded with the significant decline in both saproxylic beetle species and abundances (Fig. 6). Forest treatment operations (including prescribed burns that leave patches of unburned areas) that preserve snags, stumps and logs will provide more favorable habitat for saproxylic species.

## Conclusion and Management Implications

The results of this case study indicated that fuels reduction operations involving tree thinning and prescribed cool-season broadcast slash fire in mixed-conifer forests of New Mexico affected the surface-active beetle community by shifting abundances of common species and creating a distinct beetle assemblage in the more open, grassy subcanopy habitat. While exhibiting significant effects, the beetle community’s response to the treatments involved the addition of 2 common colonizing beetle species, and the local extirpation of 3 common forest beetle species (all of which continued to reside in the adjacent control forest). In addition, the beetle community trophic structure remained nearly the same, although saproxylic species occurrences and abundances decreased following thinning and burning compared to the untreated forest. Taken altogether, these forest restoration treatments had much milder impacts than stand-replacement wildfires and clear-cutting. For example, Clayton (2002) found stand-replacement wildfire in mixed-conifer forests in northern Utah resulted in changes in the surface-active beetle community 1 to 3 years after fire, which resulted in a Jaccard’s similarity index between burned and unburned forest of 0.241, whereas our post-fire similarity value averaged 0.388 1 to 3 years after prescribed fire. In addition, Clayton (2002) found that clear-cutting resulted in a similarity index of 0.344 1 to 4 years after cutting, while our post-thinning similarity averaged 0.444 1 to 2 years post-cutting. In northern Arizona, Chen et al. (2006) reported that thinning and burning in ponderosa pine forests resulted in smaller beetle community changes from those of control forests than in stands subjected to stand-replacement wildfires. Given the benefits of fuel reductions in preventing high-severity, stand-replacement forest fires, such treatments should be continued as an effective means of restoring stand structure and natural low-intensity fire regimes. Cool-season prescribed fires appear to have few negative direct effects on surface-active beetles, and should also be continued as part of fire management programs. Some coarse woody debris, snags, and stumps should be left on treated sites to support saproxylic beetle species. Maintaining a mosaic of forest habitat patches in different seral stages should promote maximal beetle diversity at the landscape level.

Finally, a question often arises when designing forest management monitoring programs as to the level of taxonomic detail necessary for arthropod identifications. Some studies have only reported to the order-level, and produced useful insights (e.g. de Oliveira et al. 2020, Common et al. 2025), while others focused on family-level identifications (e.g. Collett 2000, Rieske and Buss 2001, Yu et al. 2006a,b, Elia et al. 2012). Likely due to taxonomic familiarity, numerous researchers have proposed particular families within the Coleoptera as “bioindicator species” for assessing or monitoring forest management practices with respect to ecosystem conservation. Foremost among them (by sheer numbers of publications) in coniferous forests are the Carabidae (reviewed by Koivula 2011, Stočes and Šipoš 2025) and the Staphylinidae (Michaels 2007, Klimaszewski et al. 2018); however, other taxa from different forest types have received attention due to their sensitivity to forest disturbances, including Scarabaeidae in tropical forests (Noriega et al. 2020, López-Bedoya et al. 2022), and Tenebrionidae in Australian eucalyptus forests (Michaels 2007). While individual beetle families can document changes in composition and abundances of their component species, single families are often limited in their trophic ecosystem roles. For example, carabids are nearly all predators with a few genera of omnivores, and staphylinids are generally split between predators and fungivores. We suggest that a more comprehensive assessment of ecosystem disturbance can be accomplished when using all the resident beetle species encompassing all the various trophic groups, as higher taxonomic groupings or limited family inclusions can mask important community dynamics and complexities, especially with respect to rarer species (see also Grimbacher et al. 2008, de Oliveira et al. 2020, Miller-ter Kuile et al. 2025). In addition, other arthropod taxa have been shown to contribute unique perspectives to monitoring programs, including spiders (Pearce and Venier 2006, Príeto-Benítez and Méndez 2011) and grasshoppers/crickets (Orthoptera; reviewed in Parmenter et al. 2026); these taxa should be included in forest management assessments when logistically feasible.

## Supplementary Material

nvag098_Supplementary_Data

## Data Availability

Data are available to the public at the following National Park Service’s Integrated Resource Management Applications (IRMA) DataStore websites: Coleoptera dataset: https://irma.nps.gov/DataStore/Reference/Profile/2318131 Vegetation dataset: https://irma.nps.gov/DataStore/Reference/Profile/2314768 Meteorological dataset: https://irma.nps.gov/DataStore/Reference/Profile/2298922
