# Supplementary material for: Response of the surface-active beetle community (Coleoptera) to mixed-conifer forest restoration thinning and burning treatments in northern New Mexico, USA
Source: Environ Entomol. 2026 Sep 17;55(5):nvag098. doi: 10.1093/ee/nvag098 (PMC13585400; doi:10.1093/ee/nvag098)
Supplement: Supplementary file 1 [file nvag098_supplementary_data.pdf]

Appendix A. Supplemental Materials.

Response of the surface-active beetle community (Coleoptera) to mixed-conifer forest restoration thinning and burning treatments in northern New Mexico, USA

Robert R. Parmenter[1,2,*], Richard J. Buss[1,2], Benjamin P. Stout[1], Sarah M. Hall[1,†], and Orrin B. Myers[3]

[1] Valles Caldera National Preserve, National Park Service, Jemez Springs, NM 87025 USA
[2] Museum of Southwestern Biology, Division of Arthropods, Department of Biology, University of New Mexico, Albuquerque, NM 87131 USA
[3] Department of Family and Community Medicine, University of New Mexico, Albuquerque, NM 87131 USA

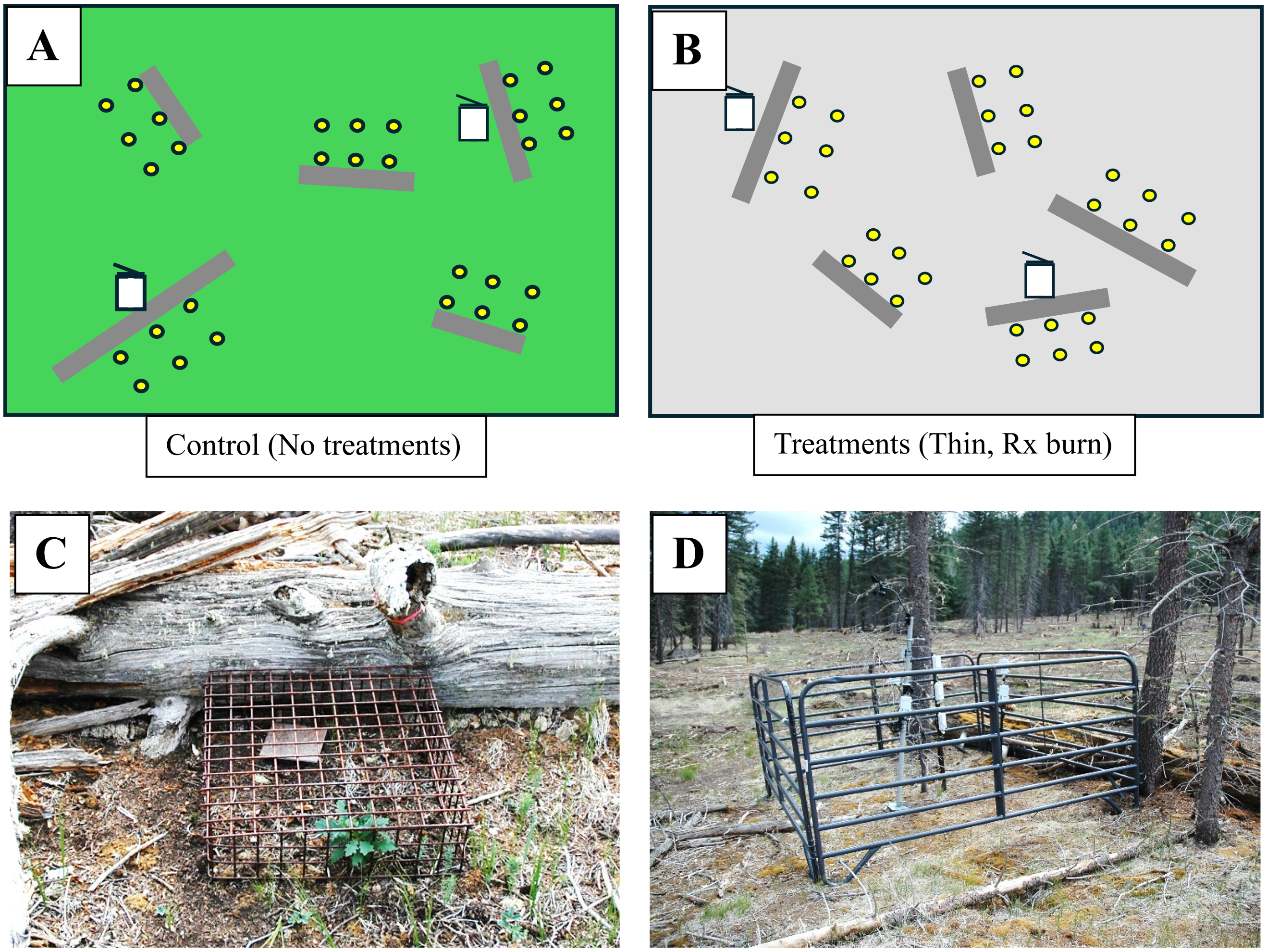

Fig. S1. Study site map and photos. (A, B) Schematic layout (not to scale) of experimental design of arthropod pitfall traps ( ) arrayed adjacent to and away from logs ( ) in control and treated mixed-conifer forest areas within the Cerro Seco Unit 5 project area in Valles Caldera National Preserve, NM. Pitfall traps were operational for 4 months in each year (June, July, August, September), and sampled for 6 years: 2017 – Pre-treatment; 2018-2019 – Post-thinning; 2020-2022 – Post-Rx fire. = On-site meteorological stations. (C) Photograph of arthropod pitfall trap next to log, showing protective steel-mesh cage to prevent trap disturbance by elk, bears and other wildlife. Steel-mesh was 5 cm x 5 cm, and the cage was secured into the ground with two steel rebar posts; mesh cover was hinged to open for access to traps, and wired shut while trap was operating. (D) Photograph of on-site meteorological station next to a log in the post-thin treatment area; steel fence panels prevented damage from elk. Photos: R. R. Parmenter. Figure plate from Parmenter et al. (2026), with permission.

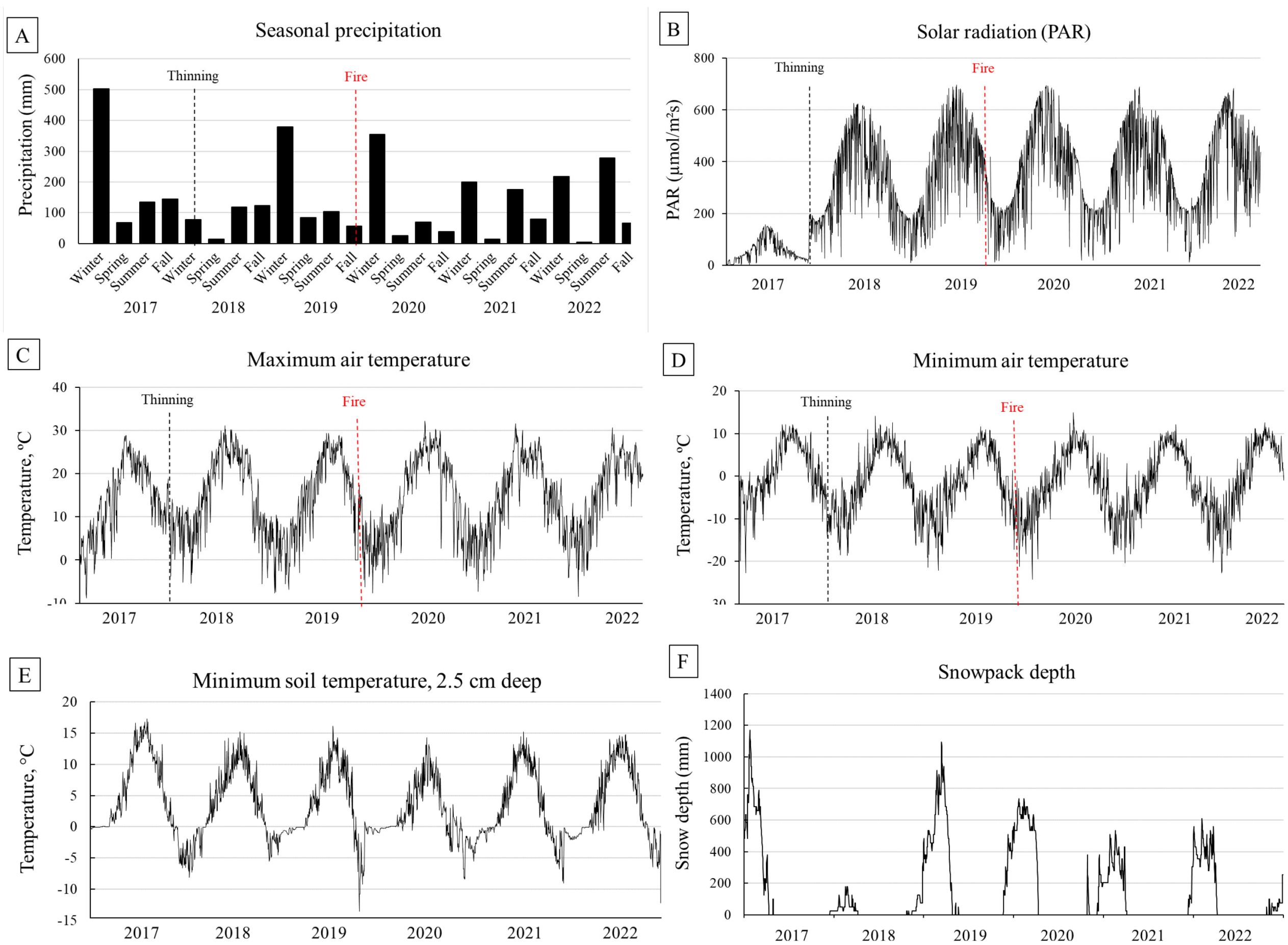

Fig. S2. Meteorological conditions during the forest restoration study (2017-2022) on the Cerro Seco Unit 5 project site in Valles Caldera National Preserve, NM. (A) Seasonal precipitation; (B) Photosynthetically-Active Radiation (PAR); (C) daily maximum air temperature; (D) daily minimum air temperature; (E) minimum soil temperature at 2.5 cm deep; (F) snowpack depth. Precipitation, PAR and air temperature data (A–D) were from the Cerro Seco Unit 5 study site (see Parmenter and Losleben 2023); soil temperature data (E) were from the Valle San Antonio RAWS site (https://wrcc.dri.edu/vallescaldera/); snowpack depth data (F) were from the Garita Peak SNOTEL station (https://wcc.sc.egov.usda.gov/nwcc/site?sitenum=1173). Figure plate from Parmenter et al. (2026), with permission.

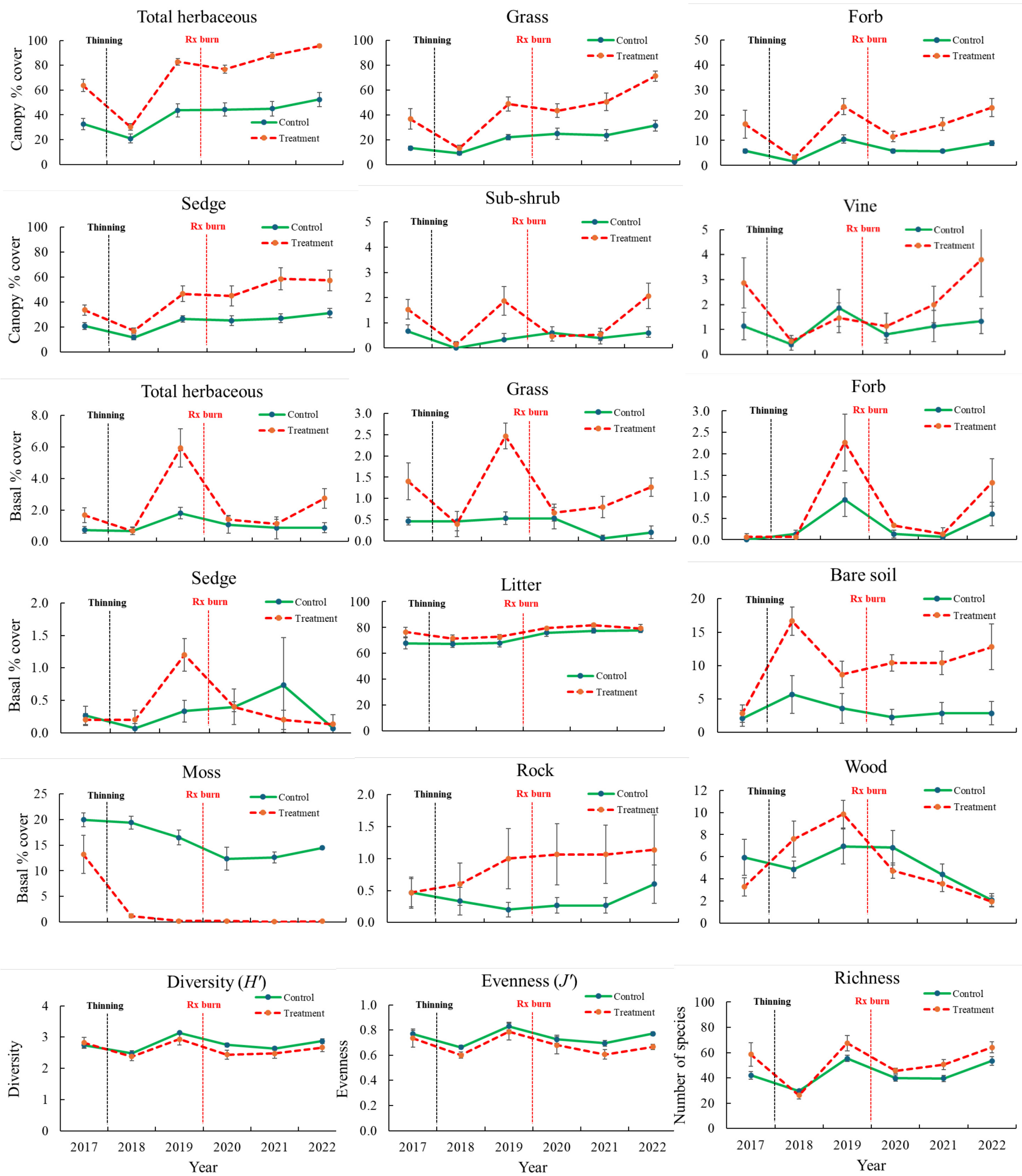

Fig. S3. Results of vegetation measures on the Cerro Seco Unit 5 project site, Valles Caldera National Preserve, NM. Top two rows are canopy cover of vegetation; next three rows are basal cover; bottom row shows plant Shannon-Wiener diversity, evenness and species richness. Points are means of 5 sample sites, error bars show SE. Figure plate from Parmenter et al. (2026), with permission.

Table S1. Split-plot Analysis of Variance (ANOVA) results tables for vegetation and forest-floor percent cover variables during 2017-2022 on the Cerro Seco Unit 5 project site in Valles Caldera National Preserve, NM. See Fig. S2 for variables' graphic results. From Parmenter et al. (2026), with permission.

Variable: Total herbaceous canopy cover

**Source**	***df***	***SS***	***MS***	***F***	***P***
Site	4	893.2	223.3		
Treatment	1	16368.0	16368.0	59.39	**0.0015**
Error Site*Treatment	4	1102.5	275.6		
Year	5	14915.0	2983.0	65.72	**0.0000**
Treatment*Year	5	2010.6	402.1	8.86	**0.0000**
Error Site*Treatment*Year	40	1815.5	45.4		
Total	59	37104.8			

Variable: Total grass canopy cover

**Source**	***df***	***SS***	***MS***	***F***	***P***
Site	4	1649.1	412.27		
Treatment	1	8073.6	8073.60	17.44	**0.0140**
Error Site*Treatment	4	1851.8	462.94		
Year	5	8931.8	1786.36	79.83	**0.0000**
Treatment*Year	5	1743.5	348.71	15.58	**0.0000**
Error Site*Treatment*Year	40	895.1	22.38		
Total	59	23144.9			

Variable: Total forb canopy cover

**Source**	***df***	***SS***	***MS***	***F***	***P***
Site	4	328.91	82.23		
Treatment	1	1022.31	1022.31	14.65	**0.0187**
Error Site*Treatment	4	279.16	69.79		
Year	5	1085.59	217.12	21.02	**0.0000**
Treatment*Year	5	202.39	40.48	3.92	**0.0055**
Error Site*Treatment*Year	40	413.08	10.33		
Total	59	3331.44			

Variable: Total sedge canopy cover

**Source**	***df***	***SS***	***MS***	***F***	***P***
Site	4	1911.8	477.95		
Treatment	1	5568.1	5568.07	23.33	**0.0085**
Error Site*Treatment	4	954.6	238.65		
Year	5	6191.5	1238.29	20.21	**0.0000**
Treatment*Year	5	1081.9	216.38	3.53	**0.0097**
Error Site*Treatment*Year	40	2450.5	61.26		
Total	59	18158.3			

Variable: Total sub-shrub canopy cover

**Source**	***df***	***SS***	***MS***	***F***	***P***
Site	4	2.9556	0.73889		
Treatment	1	6.6667	6.66667	23.23	**0.0085**
Error Site*Treatment	4	1.1481	0.28704		
Year	5	11.7778	2.35556	10.83	**0.0000**
Treatment*Year	5	6.6000	1.32000	6.07	**0.0003**
Error Site*Treatment*Year	40	8.6963	0.21741		
Total	59	37.8444			

Variable: Total vine canopy cover

**Source**	***df***	***SS***	***MS***	***F***	***P***
Site	4	24.252	6.0630		
Treatment	1	10.980	10.9796	2.75	0.1728
Error Site*Treatment	4	15.993	3.9981		
Year	5	27.631	5.5263	3.57	**0.0092**
Treatment*Year	5	14.343	2.8685	1.85	0.1246
Error Site*Treatment*Year	40	61.933	1.5483		
Total	59	155.131			

Variable: Total herbaceous basal cover

**Source**	***df***	***SS***	***MS***	***F***	***P***
Site	4	5.158	1.2895		
Treatment	1	23.655	23.6548	60.33	**0.0015**
Error Site*Treatment	4	1.568	0.3921		
Year	5	66.989	13.3979	18.25	**0.0000**
Treatment*Year	5	30.413	6.0827	8.28	**0.0000**
Error Site*Treatment*Year	40	29.371	0.7343		
Total	59	157.154			

Variable: Total grass basal cover

**Source**	***df***	***SS***	***MS***	***F***	***P***
Site	4	0.4349	0.10873		
Treatment	1	9.3405	9.34050	27.44	**0.0063**
Error Site*Treatment	4	1.3614	0.34036		
Year	5	8.1643	1.63286	7.04	**0.0001**
Treatment*Year	5	6.4334	1.28667	5.55	**0.0006**
Error Site*Treatment*Year	40	9.2758	0.23189		
Total	59	35.0102			

Variable: Total forb basal cover

Source	*df*	*SS*	*MS*	*F*	*P*
Site	4	2.4444	0.61111		
Treatment	1	2.4000	2.40000	6.00	0.0705
Error Site*Treatment	4	1.6000	0.40000		
Year	5	20.0667	4.01333	13.09	**0.0000**
Treatment*Year	5	3.7778	0.75556	2.46	**0.0489**
Error Site*Treatment*Year	40	12.2667	0.30667		
Total	59	42.5556			

Variable: Total sedge basal cover

Source	*df*	*SS*	*MS*	*F*	*P*
Site	4	0.4741	0.11852		
Treatment	1	0.0907	0.09074	0.27	0.6330
Error Site*Treatment	4	1.3630	0.34074		
Year	5	3.1278	0.62556	2.25	0.0683
Treatment*Year	5	2.5648	0.51296	1.84	0.1266
Error Site*Treatment*Year	40	11.1407	0.27852		
Total	59	18.7611			

Variable: Total litter ground cover

Source	*df*	*SS*	*MS*	*F*	*P*
Site	4	547.44	136.861		
Treatment	1	327.02	327.024	5.39	0.0810
Error Site*Treatment	4	242.72	60.681		
Year	5	1096.74	219.349	16.45	**0.0000**
Treatment*Year	5	57.84	11.567	0.87	0.5114
Error Site*Treatment*Year	40	533.41	13.335		
Total	59	2805.19			

Variable: Total bare soil ground cover

Source	*df*	*SS*	*MS*	*F*	*P*
Site	4	167.46	41.864		
Treatment	1	748.84	748.840	10.13	**0.0334**
Error Site*Treatment	4	295.62	73.905		
Year	5	398.55	79.710	10.57	**0.0000**
Treatment*Year	5	173.16	34.633	4.59	**0.0021**
Error Site*Treatment*Year	40	301.70	7.542		
Total	59	2085.33			

Variable: Total moss ground cover

**Source**	***df***	***SS***	***MS***	***F***	***P***
Site	4	20.39	5.10		
Treatment	1	2683.95	2683.95	155.64	**0.0002**
Error Site*Treatment	4	68.98	17.24		
Year	5	768.13	153.63	18.61	**0.0000**
Treatment*Year	5	196.09	39.22	4.75	**0.0017**
Error Site*Treatment*Year	40	330.18	8.25		
Total	59	4067.73			

Variable: Total rock ground cover

**Source**	***df***	***SS***	***MS***	***F***	***P***
Site	4	20.9915	5.24789		
Treatment	1	4.2792	4.27925	0.79	0.4239
Error Site*Treatment	4	21.6196	5.40490		
Year	5	1.1375	0.22750	1.18	0.3353
Treatment*Year	5	1.4223	0.28445	1.48	0.2188
Error Site*Treatment*Year	40	7.7022	0.19256		
Total	59	57.1524			

Variable: Total wood ground cover

**Source**	***df***	***SS***	***MS***	***F***	***P***
Site	4	95.146	23.7864		
Treatment	1	18.392	18.3921	1.04	0.3664
Error Site*Treatment	4	71.032	17.7581		
Year	5	260.719	52.1437	10.26	**0.0000**
Treatment*Year	5	90.162	18.0324	3.55	**0.0095**
Error Site*Treatment*Year	40	203.329	5.0832		
Total	59	738.780			

Variable: Plant community diversity (*H'*)

**Source**	***df***	***SS***	***MS***	***F***	***P***
Site	4	1.24812	0.31203		
Treatment	1	0.34105	0.34105	1.66	0.2668
Error Site*Treatment	4	0.82085	0.20521		
Year	5	2.29074	0.45815	25.41	**0.0000**
Treatment*Year	5	0.21680	0.04336	2.40	0.0536
Error Site*Treatment*Year	40	0.72131	0.01803		
Total	59	5.63887			

Variable: Plant community evenness (*J'*)

**Source**	***df***	***SS***	***MS***	***F***	***P***
Site	4	0.08894	0.02223		
Treatment	1	0.05947	0.05947	4.88	0.0917
Error Site*Treatment	4	0.04873	0.01218		
Year	5	0.21291	0.04258	8.82	**0.0000**
Treatment*Year	5	0.00980	0.00196	0.41	0.8418
Error Site*Treatment*Year	40	0.19304	0.00483		
Total	59	0.61288			

Variable: Plant community species richness

**Source**	***df***	***SS***	***MS***	***F***	***P***
Site	4	101.2	25.31		
Treatment	1	1161.6	1161.60	31.14	**0.0051**
Error Site*Treatment	4	149.2	37.31		
Year	5	789.9	157.99	0.68	0.6446
Treatment*Year	5	1010.6	202.12	0.86	0.5138
Error Site*Treatment*Year	40	9359.1	233.98		
Total	59	12571.7			

Table S2. List of beetle (Coleoptera) species recorded during 2017-2022 on the Cerro Seco Unit 5 project site in Valles Caldera National Preserve, NM. (-) = no data available for trophic position.

	Number of specimens		Trophic position		Sapro-xylic
Family/*Genus species*	Control	Treatment	Adult	Larva	
Agyrtidae					
Apteroloma sp. #1	2	0	Detritivore	-	
Anthicidae					
Anthicus hastatus Casey, 1895	1	3	Omnivore	Omnivore	
Anthicus basimacula Champion, 1890	0	1	Omnivore	Omnivore	
Notoxus anchora Hentz, 1827	2	8	Omnivore	Omnivore	
Bothrideridae					
Oxylaemus californicus Crotch, 1874	0	1	Fungivore	Fungivore	X
Buprestidae					
Anthaxia sp. #1	3	1	Herbivore	Herbivore	
Phaenops drummondi (Kirby, 1837)	0	1	Herbivore	Herbivore	
Byrrhidae					
Byrrhus cyclophorus Kirby, 1837	0	1	Herbivore	Herbivore	
Byrrhus eximius LeConte, 1850	9	10	Herbivore	Herbivore	
Byrrhus geminatus LeConte, 1854	1	0	Herbivore	Herbivore	
Byrrhus kirbyi LeConte, 1854	1	9	Herbivore	Herbivore	
Curimopsis echinata (LeConte, 1850)	6	4	Herbivore	Herbivore	
Cytilus alternatus (Say, 1825)	1	0	Herbivore	Herbivore	
Cytilus mimicus Casey, 1912	1	0	Herbivore	Herbivore	
Morychus oblongus (LeConte, 1857)	1	0	Herbivore	Herbivore	
Porcinolus undatus (Melsheimer, 1844)	1	3	Herbivore	Herbivore	
Cantharidae					
Dichelotarsus sp. #1	6	0	Omnivore	Predators	
Malthodes sp. #1	7	2	Omnivore	Predators	
Silis sp. #1	9	1	Omnivore	Predators	
Silis pallida Mannerheim, 1843	0	1	Omnivore	Predators	
Carabidae					
Agonum cupreum Dejean, 1831	0	5	Predator	Predator	
Agonum cupripenne (Say, 1823)	1	13	Predator	Predator	
Agonum fossigerum Dejean, 1828	0	1	Predator	Predator	
Agonum muiri Liebherr, 1984	0	1	Predator	Predator	
Agonum placidum (Say, 1823)	2	23	Omnivore	Predator	
Agonum texanum LeConte, 1878	1	1	Predator	Predator	
Amara convexa LeConte, 1848	2	6	Predator	Predator	
Amara ellipsis (Casey, 1918)	4	256	Predator	Predator	
Amara latior (Kirby, 1937)	0	3	Predator	Predator	

Amara littoralis Mannerheim, 1843	4	76	Omnivore	Predator	
Amara obesa (Say, 1823)	0	2	Predator	Predator	
Apristus sp. #1	0	1	Predator	Predator	
Bembidion mutatum Gemminger & Harold, 1868	0	17	Predator	Predator	
Bembidion sp. #2	3	10	Predator	Predator	
Calathus advena (LeConte, 1848)	3,783	387	Predator	Predator	
Calathus ingratus DeJean, 1828	432	235	Predator	Predator	
Calosoma calidum (Fabricius, 1775)	1	0	Predator	Predator	
Calosoma peregrinator Guérin-Méneville, 1844	1	4	Predator	Predator	
Carabus taedatus Fabricius, 1787	6,996	4,455	Predator	Predator	
Cicindela longilabris Say, 1824	0	154	Predator	Predator	
Cicindela purpurea cimarrona LeConte, 1868	0	13	Predator	Predator	
Cymindis cribricollis Dejean, 1831	58	68	Predator	Predator	
Dyschirius sp. #1	5	8	Predator	Predator	
Harpalus animosus Casey, 1924	152	1,314	Omnivore	Predator	
Harpalus ellipsis LeConte, 1847	3	27	Omnivore	Predator	
Harpalus nigritarsis C.R. Sahlberg, 1827	17	128	Omnivore	Predator	
Harpalus opacipennis (Haldemann, 1843)	4	13	Omnivore	Predator	
Harpalus pensylvanicus (DeGeer, 1774)	0	1	Omnivore	Predator	
Harpalus somnulentus Dejean, 1829	10	94	Omnivore	Predator	
Notiophilus directus Casey, 1920	113	25	Predator	Predator	
Notiophilus semistriatus Say, 1823	36	27	Predator	Predator	
Pterostichus adstrictus Eschscholtz, 1823	4,144	3,097	Predator	Predator	
Pterostichus restrictus (Casey, 1918)	3,951	1,130	Predator	Predator	
Rhadine nivalis (G. Horn, 1881)	367	720	Predator	Predator	
Scaphinotus snowi (LeConte, 1881)	9	13	Predator	Predator	
Selenophorus planipennis LeConte, 1847	0	2	Predator	Predator	
Sericoda bembidioides Kirby, 1837	0	98	Predator	Predator	
Sericoda quadripunctata (DeGeer, 1774)	39	192	Predator	Predator	
Syntomus americanus (Dejean, 1831)	2	8	Predator	Predator	
Synuchus dubius (LeConte, 1854)	100	127	Omnivore	Predator	
Cerambycidae					
Acmaeops proteus (Kirby in Richardson, 1837)	2	1	Herbivore	Herbivore	X
Calloides nobilis (Harris, 1837)	0	2	Herbivore	Herbivore	X
Grammoptera haematites (Newman, 1841)	0	1	Herbivore	Herbivore	X
Lepturobosca nigrolineata (Bland, 1865)	1	2	Herbivore	Herbivore	X
Megasemum asperum (LeConte, 1854)	1	0	Herbivore	Herbivore	X
Monochamus clamator LeConte, 1852	0	1	Herbivore	Herbivore	X
Monochamus scutellatus (Say, 1824)	0	1	Herbivore	Herbivore	X
Pachyta lamed (Linnaeus, 1758)	0	1	Herbivore	Herbivore	X
Phymatodes maculicollis LeConte, 1878	1	0	Herbivore	Herbivore	X

Prionus heroicus Semenov, 1907	0	1	Herbivore	Herbivore	X
Tragosoma harrisii LeConte, 1851	1	14	Herbivore	Herbivore	X
Xestoleptura cockerelli Fall, 1970	3	7	Herbivore	Herbivore	X
Xylotrechus bowditchi Hopping, 1928	1	1	Herbivore	Herbivore	X
Chrysomelidae					
Altica torquata J.L. LeConte, 1858	1	1	Herbivore	Herbivore	
Chaetocnema sp. #1	0	1	Herbivore	Herbivore	
Disonycha semicarbonata (LeConte, 1859)	0	8	Herbivore	Herbivore	
Glyptina sp. #1	1	0	Herbivore	Herbivore	
Graphops sp. #1	0	1	Herbivore	Herbivore	
Longitarsus sp. #1	0	7	Herbivore	Herbivore	
Phyllotreta constricta E. Smith, 1985	1	0	Herbivore	Herbivore	
Phyllotreta sp. #1	12	33	Herbivore	Herbivore	
Phyllotreta sp. #2	0	2	Herbivore	Herbivore	
Psylliodes sp. #1	0	4	Herbivore	Herbivore	
Syneta sp. #1	2	0	Herbivore	Herbivore	
Ciidae					
Cis creberrimus Mellié, 1848	0	1	Fungivore	Fungivore	
Orthocis punctatus (Mellié, 1848)	1	1	Fungivore	Fungivore	
Cleridae					
Enoclerus lecontei (Wolcott, 1910)	0	1	Predator	Predator	
Enoclerus moestus (Klug, 1842)	0	2	Predator	Predator	
Phyllobaenus subfasciatus (LeConte, 1866)	0	1	Predator	Predator	
Thanasimus undatulus (Say, 1835)	1	0	Predator	Predator	
Coccinellidae					
Anatis lecontei Casey, 1899	1	0	Predator	Predator	
Coccinella septempunctata Linnaeus, 1758	0	11	Predator	Predator	
Exochomus aethiops (Bland, 1864)	0	5	Predator	Predator	
Exochomus townsendi Casey, 1908	0	23	Predator	Predator	
Hippodamia convergens Guerin, 1842	3	98	Predator	Predator	
Hippodamia parenthesis (Say, 1824)	1	124	Predator	Predator	
Hyperaspis inflexa Casey, 1899	0	1	Predator	Predator	
Myzia interrupta (Casey, 1899)	1	0	Predator	Predator	
Psyllobora vigintimaculata (Say, 1824)	0	1	Predator	Predator	
Scymnus barberi Gordon, 1976	0	2	Predator	Predator	
Corylophidae					
Arthrolips sp. #1	0	1	Fungivore	Fungivore	
Clypastraea sp. #1	0	1	Fungivore	Fungivore	
Orthoperus brunneus (Casey, 1900)	4	0	Fungivore	Fungivore	
Cryptophagidae					
Atomaria sp. #1 (subg. *Atomaria*)	995	1,741	Fungivore	Fungivore	

Atomaria sp. #2 (subg. *Anchicera*)	158	22	Fungivore	Fungivore	
Caenoscelis sp. #1	6	10	Fungivore	Fungivore	
Cryptophagus jemezmontanus Esser, 2018	7	1	Fungivore	Fungivore	
Cryptophagus latens Woodroffe & Coombs, 1961	3	0	Fungivore	Fungivore	
Cryptophagus pilosus Gyllenhal, 1827	1,200	213	Fungivore	Fungivore	
Cryptophagus porrectus Casey, 1900	8	1	Fungivore	Fungivore	
Cryptophagus redondoensis Esser, 2018	4	0	Fungivore	Fungivore	
Cryptophagus tuberculosus Mäklin, 1853	348	412	Fungivore	Fungivore	
Henoticus californicus (Mannhereim, 1843)	1	0	Fungivore	Fungivore	
Henoticus serratus (Gyllenhal, 1808)	1	2	Fungivore	Fungivore	
Myrmedophila americana (LeConte, 1879)	3	1	Fungivore	Fungivore	
Curculionidae					
Ceutorhynchus sp. #1	0	7	Herbivore	Herbivore	
Ceutorhynchus sp. #2	0	1	Herbivore	Herbivore	
Ceutorhynchus sp. #3	1	0	Herbivore	Herbivore	
Cossonus sp. #1	0	1	Herbivore	Herbivore	X
Crypturgus borealis Swaine, 1917	1	0	Herbivore	Herbivore	X
Dendroctonus pseudotsugae Hopkins, 1909	2	2	Herbivore	Herbivore	X
Dendroctonus valens LeConte, 1860	0	1	Herbivore	Herbivore	X
Hylastes gracilis LeConte, 1869	28	38	Herbivore	Herbivore	X
Ips pini (Say, 1826)	5	0	Herbivore	Herbivore	X
Magdalis sp. #1	1	0	Herbivore	Herbivore	X
Otiorhynchus ovatus (Linnaeus, 1758)	46	77	Herbivore	Herbivore	
Panscopus coloradensis Van Dyke, 1936	0	6	Herbivore	Herbivore	
Pissodes sp. #1	1	0	Herbivore	Herbivore	
Pityophthorus sp. #1	0	1	Herbivore	Herbivore	X
Pityophthorus sp. #2	1	0	Herbivore	Herbivore	X
Pseudorimus orbicollis Van Dyke, 1934	11	25	Herbivore	Herbivore	
Rhyncolus sp. #1	10	5	Herbivore	Herbivore	X
Thricolepis inornata Horn, 1876	39	5	Herbivore	Herbivore	
Trichalophus sp. #1	3	1	Herbivore	Herbivore	
Trypodendron lineatum (Olivier, 1795)	2	0	Herbivore	Herbivore	X
Dermestidae					
Anthrenus coloratus Reitter, 1881	1	1	Detritivore	Detritivore	
Dermestes talpinus Mannerheim, 1843	0	1	Detritivore	Detritivore	
Megatoma variegata (Horn, 1875)	8	0	Herbivore	Herbivore	
Elateridae					
Ampedus n.sp.	1	1	Herbivore	Herbivore	X
Ampedus sp. #2	2	0	Herbivore	Herbivore	X
Athous arizonicus Van Dyke, 1932	29	25	Herbivore	Herbivore	
Athous sp. #2	36	39	Herbivore	Herbivore	

Hemicrepidius carbonatus (LeConte, 1860)	0	1	Herbivore	Herbivore	
Laneganus sp. #1	18	1	Herbivore	Herbivore	
Limonius aeger LeConte, 1853	18	9	Herbivore	Herbivore	
Megapenthes sp. #1	0	1	Herbivore	Herbivore	
Melanotus castanipes (Paykull, 1800)	10	6	Herbivore	Herbivore	
Selatosomus sp. #1	0	1	Herbivore	Herbivore	
Stropenron pudica (Brown, 1936)	18	8	Herbivore	Herbivore	
Stropenron sp. #2	219	15	Herbivore	Herbivore	
Endomychidae					
Aphorista morosa (LeConte 1859)	10	42	Fungivore	Fungivore	
Erotylidae					
Cypherotylus californicus (Lacordaire, 1842)	0	3	Fungivore	Fungivore	
Triplax mesosternalis Schaeffer, 1905	1	0	Fungivore	Fungivore	
Eucinetidae					
Euscaphurus spinipes Vit, 1995	2	1	Fungivore	Fungivore	
Eucnemidae					
Epiphanis cornutus (Eschscholtz, 1829)	1	0	Fungivore	Fungivore	X
Geotrupidae					
Odonteus obesus (LeConte, 1859)	0	5	Detritivore	Detritivore	
Histeridae					
Euspilotus sp. #1	0	2	Predator	Predator	
Hister furtivus J.E. LeConte, 1860	30	43	Predator	Predator	
Plegaderus sayi Marseul, 1856	0	1	Predator	Predator	
Psiloscelis subopaca (LeConte, 1863)	2	2	Predator	Predator	
Saprinus (*Xerosaprinus*) sp. #1	9	31	Predator	Predator	
Latridiidae					
Cartodere (*Aridius*) *australica* Belon, 1887	6	1	Fungivore	Fungivore	
Corticaria sp. #1	1,094	723	Fungivore	Fungivore	
Corticarina sp. #1	8	16	Fungivore	Fungivore	
Enicmus sp. #1	2	4	Fungivore	Fungivore	
Latridius sp. #1	64	27	Fungivore	Fungivore	
Melanophthalma sp. #1	4	0	Fungivore	Fungivore	
Leiodidae					
Agathidium angulare Mannerheim, 1852	12	5	Fungivore	Fungivore	
Agathidium concinnum Mannerheim, 1852	15	1	Fungivore	Fungivore	
Agathidium depressum Fall, 1934	1	0	Fungivore	Fungivore	
Agathidium estriatum Horn, 1880	6	1	Fungivore	Fungivore	
Agathidium omissum Fall, 1934	4	0	Fungivore	Fungivore	
Agathidium pulchrum LeConte, 1853	154	8	Fungivore	Fungivore	
Anisotoma sp. #1	3	6	Fungivore	Fungivore	
Anogdus superans (Fall, 1910)	0	1	Fungivore	Fungivore	

Catops sp. #1	74	79	Fungivore	Fungivore	
Colon liebecki Wickham, 1902	1	0	Fungivore	Fungivore	
Colon sp. #2	1	7	Fungivore	Fungivore	
Dissochaetus arizonensis Hatch, 1933	0	1	Fungivore	Fungivore	
Hydnobius laticeps Notman, 1920	1	0	Fungivore	Fungivore	
Hydnobius longidens LeConte, 1879	2	22	Fungivore	Fungivore	
Hydnobius pumilus LeConte, 1879	0	1	Fungivore	Fungivore	
Hydnobius substriatus LeConte, 1863	0	9	Fungivore	Fungivore	
Liocyrtusa nigriclavis (Hlisnikovsky, 1967)	0	2	Fungivore	Fungivore	
Lionothus forticornis Daffner, 1989	1	0	Fungivore	Fungivore	
Macrohydnobius crestonensis (Hatch, 1957)	1	0	Fungivore	Fungivore	
Ptomaphagus sp. #1	37	29	Fungivore	Fungivore	
Sogda obtusa (J. L. LeConte, 1879)	1	1	Fungivore	Fungivore	
Lycidae					
Dictyoptera coccinata (Say, 1835)	7	1	Herbivore	Fungivore	X
Lygistopterus perfacetus (Say 1825)	0	2	Herbivore	Fungivore	X
Melandryidae					
Scotochroa sp. #1	1	1	Fungivore	Fungivore	X
Serropalpus substriatus Haldeman, 1848	0	2	Fungivore	Fungivore	X
Meloidae					
Epicauta sp. #1	0	5	Herbivore	Parasitoid	
Melyridae					
Attalus sp. #1	2	0	Omnivore	Omnivore	
Monotomidae					
Rhizophagus dimidiatus Mannerheim, 1843	3	1	Predator	Predator	X
Rhizophagus remotus LeConte, 1866	14	2	Omnivore	Omnivore	X
Mordellidae					
Mordellistena sp. #1	0	1	Herbivore	Herbivore	
Mycetophagidae					
Neotriphyllus confusus (Horn, 1878)	2	1	Fungivore	Fungivore	
Mycteridae					
Lacconotus pinicola Horn, 1879	0	1	Herbivore	-	
Nitidulidae					
Carpophilus mutilatus Erichson, 1843	0	1	Fungivore	Fungivore	
Epuraea alternans (Grouvelle, 1913)	239	72	Fungivore	Fungivore	
Epuraea truncatella Mannerheim, 1846	1	1	Fungivore	Fungivore	
Epuraea umbrosa Horn, 1879	4	0	Fungivore	Fungivore	
Epuraea unicolor (Olivier, 1790)	1	0	Fungivore	Fungivore	
Glischrochilus vittatus (Say, 1835)	1	0	Predator	-	X
Omosita discoidea (Fabricius, 1775)	3	1	Fungivore	Fungivore	
Pocadius basalis Schaeffer, 1911	0	2	Fungivore	Fungivore	

Soronia guttulata (LeConte, 1863)	7	6	Fungivore	Fungivore	
Thalycra sinuata Howden, 1961	4	0	Fungivore	Fungivore	
Thalycra sp. #2	3	0	Fungivore	Fungivore	
Oedemeridae					
Calopus angustus LeConte, 1851	1	1	Herbivore	Herbivore	X
Phalacridae					
Phalacrus sp. #1	0	1	Fungivore	Fungivore	
Ptiliidae					
Acrotrichis sp. #1	76	18	Fungivore	Fungivore	
Ptinidae					
Euceratocerus sp. #1	1	0	Herbivore	Herbivore	X
Hadrobregmus alternatus (Fall, 1905)	0	2	Herbivore	Herbivore	X
Pyrochroidae					
Pedilus sp. #1	0	1	Herbivore	Fungivore	
Pythidae					
Priognathus monilicornis Randall, 1838	3	0	Predator	Herbivore	X
Salpingidae					
Sphaeriestes sp. #1	0	1	Predator	Predator	X
Scarabaeidae					
Aegialia sp. #1	1	3	Detritivore	Detritivore	
Agoliinus congregatus (Mannerheim, 1853)	0	1	Detritivore	Detritivore	
Dialytodius decipiens (Horn, 1887)	1	0	Detritivore	Detritivore	
Dichelonyx sp. #1	84	20	Herbivore	Herbivore	
Diplotaxis sp. #1	2	5	Herbivore	Herbivore	
Euphoria inda (Linnaeus, 1764)	0	5	Herbivore	Herbivore	
Teuchestes fossor (Linnaeus, 1758)	0	1	Detritivore	Detritivore	
Scraptidae					
Anaspis atrata Champion, 1891	5	1	Herbivore	-	X
Anaspis rufa Say, 1825	93	24	Herbivore	-	X
Silphidae					
Heterosilpha ramosa (Say, 1823)	1	20	Detritivore	Detritivore	
Nicrophorus investigator Zetterstedt, 1824	41	67	Detritivore	Detritivore	
Thanatophilus lapponicus Herbst, 1793	0	3	Detritivore	Detritivore	
Silvanidae					
Dendrophagus cygnaei Mannerheim, 1846	12	2	Fungivore	Fungivore	X
Staphylinidae					
Acrolocha spp.	157	74	Fungivore	Predator	
Acrotona spp.	74	21	Predator	Predator	
Actizona chuskae Chandler, 1985	4	0	Predator	Predator	X
Aleochara sp. #1	27	21	Predator	Parasitoid	
Aleocharinae spp.	6,573	1,208			

Anotylus spp.	91	40	Detritivore	Detritivore	
Baeocera congener Casey, 1893	1	2	Fungivore	Fungivore	
Belonuchus sp. #1	9	6			
Belonuchus sp. #2	27	8			
Bobitobus spp.	261	198	Predator	Predator	
Bolitobius spp.	123	12	Predator	Predator	
Brachycepsis spp.	17	7	Predator	Predator	
Carpelimus spp.	28	4	Detritivore	Detritivore	
Gabrius spp.	27	27	Predator	Predator	
Homalota sp. #1	3	0	Fungivore	Fungivore	X
Leptusa sp. #1	1	0	Fungivore	Fungivore	
Lordithon spp.	154	7	Predator	Predator	
Megarthrus angulicollis Mäklin, 1852	78	19	Fungivore	Fungivore	
Micropeplus laticollis Mäklin, 1853	0	2	Fungivore	Fungivore	
Mycetoporus sp. #1	1	2	Predator	Predator	
Myrmecocephalus arizonicus (Casey, 1906)	17	12			
Neobisnius sobrinus (Erichson, 1840)	3	0			
Neobisnius sp. #2	6	5			
Olophrum sp. #1	3	1	Predator	Predator	
Omalium sp. #1	1	0	Fungivore	Fungivore	
Oxyporus occipitalis Fauvel, 1864	2	1	Fungivore	Fungivore	
Oxyporus sp. #2	0	4	Fungivore	Fungivore	
Oxytelus sp. #1	20	7	Detritivore	Detritivore	
Palporus nitidulus (Fabricius, 1781)	144	38	Predator	Predator	
Philonthus sp. #1	0	21	Predator	Predator	
Phyllodrepa sp. #1	46	39	Predator	Predator	
Phyllodrepa sp. #2	123	10	Predator	Predator	
Platydracus fossator (Gravenhorst, 1802)	2	0			
Proteinus spp.	646	38	Fungivore	Fungivore	
Pseudopsis spp.	60	27			
Quedius explanatus (J.L.LeConte, 1858)	0	1	Predator	Predator	
Quedius rusticus Smetana, 1971	12	6	Predator	Predator	
Quedius spp.	219	35	Predator	Predator	
Stenus sp. #1	20	12	Predator	Predator	
Stenus sp. #2	1	3	Predator	Predator	
Stenus sp. #3	0	1	Predator	Predator	
Tachinus sp. #1	5	5	Predator	Predator	
Tachyporus sp. #1	1	55	Fungivore	Fungivore	
Trichophya sp. #1	1	1	Fungivore	Fungivore	
Xenota spp.	122	8	Predator	Predator	

Tenebrionidae					
Blapstinus substriatus Champion, 1885	0	1	Herbivore	Herbivore	
Corticeus substriatus (LeConte, 1878)	0	1	Predator	Omnivore	X
Eleodes carbonaria (Say, 1823)	17	479	Detritivore	Herbivore	
Eleodes nigrina LeConte, 1858	0	1	Detritivore	Herbivore	
Eleodes snowii Blaisdell, 1909	369	320	Detritivore	Herbivore	
Pseudocistela pinguis (LeConte, 1859)	0	4	-	-	X
Tetratomidae					
Eustrophus tomentosus Say, 1827	2	0	Fungivore	Fungivore	
Hallomenus scapularis Melsheimer ,1846	2	7	Fungivore	Fungivore	
Hallomenus punctulatus LeConte, 1866	3	0	Fungivore	Fungivore	
Tetratoma concolor LeConte, 1879	37	4	Fungivore	Fungivore	
Trachypachidae					
Trachypachus inermis Motschulsky, 1850	146	326	Predator	Predator	
Trogidae					
Trox sonorae LeConte, 1854	7	6	Detritivore	Detritivore	
Trogossitidae					
Tenebroides tenuistriatus Fall, 1910	0	1	Predator	Predator	X
Grand Total = 55,761 specimens	35,428	20,333			

Table S3. General Linear Mixed-Model (GLMM) results tables for Coleoptera community (A) total abundances, (B) species richness, (C) diversity, and (D) evenness during forest restoration on Cerro Seco Unit 5 in Valles Caldera National Preserve, NM, 2017–2022. Calculations based on annual averages of monthly values for each trap.

A. Total abundance

Source	Numerator *df*	Denominator *df*	*F*	*P*
Year	5	350	28.46	**<0.001**
Treatment	1	10	22.15	**<0.001**
Year*Treatment	5	350	16.15	**<0.001**
Log	1	350	4.85	**0.028**
Year*Log	5	350	0.30	0.911
Treatment*Log	1	350	4.90	**0.027**
Year*Treatment*Log	5	350	1.80	0.111

Year*Treatment Comparison for adjacent years

Comparison	Difference estimate	SE	*P*	Adaptive-Holm *P*
2018-2017	-33.607	5.753	**<0.001**	**<0.001**
2019-2018	-8.816	5.753	0.126	0.126
2020-2019	17.683	5.753	**0.002**	**0.002**
2021-2020	17.613	5.753	**0.002**	**0.002**
2022-2021	-5.154	5.753	0.371	0.371

B. Species Richness

Source	Numerator *df*	Denominator *df*	*F*	*P*
Year	5	308	38.69	**<0.001**
Treatment	1	8	17.65	**0.003**
Year*Treatment	5	308	6.63	**<0.001**
Log	1	308	0.27	0.602
Year*Log	5	308	0.67	0.647
Treatment*Log	1	308	0.18	0.675
Year*Treatment*Log	5	308	1.87	0.100

Year*Treatment Comparison for adjacent years

Comparison	Difference estimate	SE	*P*	Adaptive-Holm *P*
2018-2017	-2.872	0.685	**<0.0001**	**<0.0001**
2019-2018	1.025	0.685	0.136	0.136
2020-2019	-1.547	0.685	**0.025**	**0.025**
2021-2020	1.897	0.685	**0.006**	**0.006**
2022-2021	0.572	0.685	0.404	0.404

C. Diversity

Source	Numerator *df*	Denominator *df*	*F*	*P*
Year	5	330	23.17	**<0.001**
Treatment	1	10	2.68	0.132
Year*Treatment	5	330	4.46	**<0.001**
Log	1	330	0.18	0.675
Year*Log	5	330	0.26	0.932
Treatment*Log	1	330	0.00	0.984
Year*Treatment*Log	5	330	3.01	**0.011**

<u>Year*Treatment Comparison for adjacent years</u>

Comparison	Difference estimate	SE	*P*	Adaptive-Holm *P*
2018-2017	-0.128	0.079	0.104	0.104
2019-2018	0.273	0.079	**0.001**	**0.001**
2020-2019	-0.322	0.079	**<0.0001**	**<0.0001**
2021-2020	0.065	0.079	0.408	0.408
2022-2021	0.115	0.079	0.147	0.147

D. Evenness

Source	Numerator *df*	Denominator *df*	*F*	*P*
Year	5	350	43.8	**<0.001**
Treatment	1	10	63.22	**<0.001**
Year*Treatment	5	350	12.66	**<0.001**
Log	1	350	5.79	**0.017**
Year*Log	5	350	0.14	0.983
Treatment*Log	1	350	1.97	0.161
Year*Treatment*Log	5	350	1.02	0.407

<u>Year*Treatment Comparison for adjacent years</u>

Comparison	Difference estimate	SE	*P*	Adaptive-Holm *P*
2018-2017	0.107	0.020	**<0.0001**	**<0.0001**
2019-2018	0.039	0.020	**0.053**	**0.053**
2020-2019	-0.051	0.020	**0.013**	**0.013**
2021-2020	-0.045	0.020	**0.028**	**0.028**
2022-2021	0.049	0.020	**0.016**	**0.016**

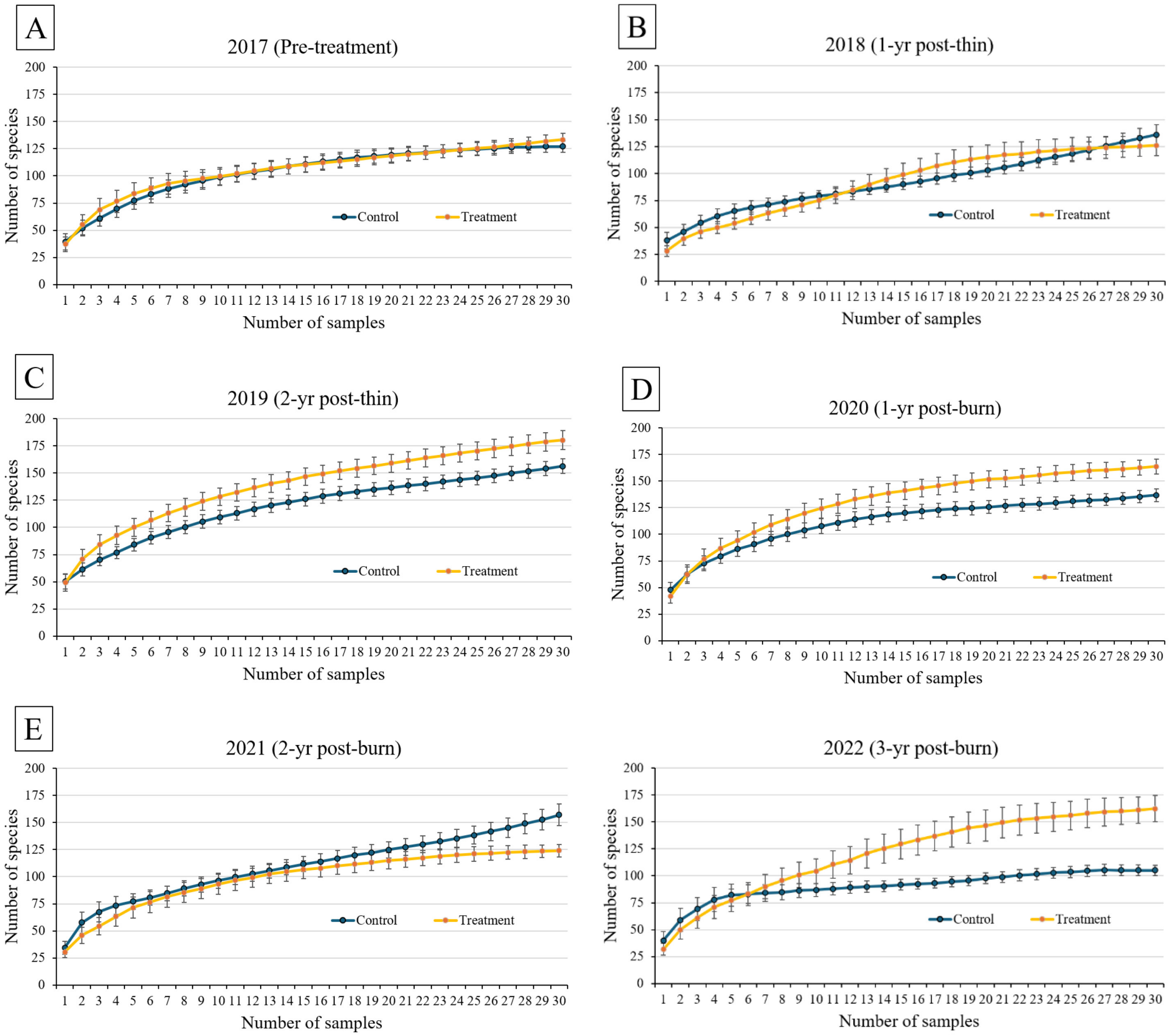

Fig. S4. Chao-1 beetle species rarefaction curves in Control and Treatment areas in different sampling years on the Cerro Seco Unit 5 project site, Valles Caldera National Preserve, NM. Curves generated in Primer-7 software. Points are means of 1,000 iterations; error bars show 95% confidence intervals.

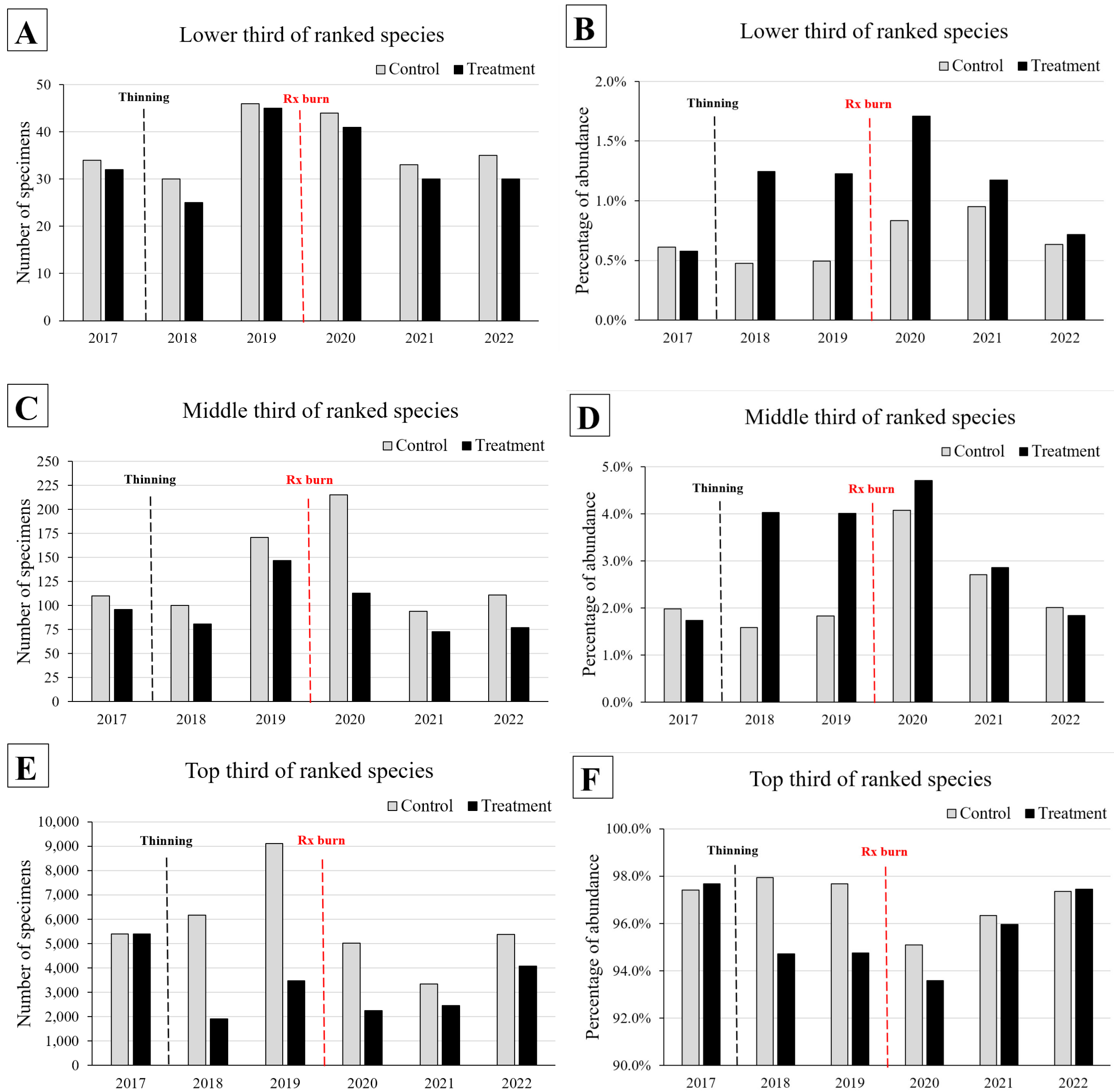

Fig. S5. Temporal changes in beetle abundances and percentage of total beetle abundances collected in Control and Treatment areas during forest thinning and burning on the Cerro Seco Unit 5 project site, Valles Caldera National Preserve, NM. Values are numbers of specimens collected (A, C, E) and percentage beetle abundances (B, D, F) in the lower third (rare species, A,B), middle third (C,D), and top third (common species, E,F) of rank-ordered species. Note that the forest thinning and burning treatment resulted in lower numbers of individuals in common species (top third of ranked species) with no major changes in rarer species abundances relative to controls; however, the reduced number of common species' abundances resulted in increased percentage abundances of rarer species.

Table S4. Beetle trophic group analysis results using the two-tailed Kolmogorov-Smirnov statistic (*KS*) for both numbers of species and numbers of individuals between treatments and through years (2017 was the pre-treatment year) on the Cerro Seco Unit 5 project area, Valles Caldera National Preserve, NM. Data shown in Fig. 5; see Appendix A, Table S2 for species' trophic group assignments. *P*-values ≤ 0.05 shown in boldface.

	Species		Individuals	
Test comparison	*KS*	*P*-value	*KS*	*P*-value
2017 Control vs. Treatment	0.07	1.000	0.06	1.000
2018 Control vs. Treatment	0.05	1.000	0.04	1.000
2019 Control vs. Treatment	0.15	0.169	0.20	**0.044**
2020 Control vs. Treatment	0.10	0.700	0.16	0.147
2021 Control vs. Treatment	0.09	0.909	0.15	0.244
2022 Control vs. Treatment	0.04	1.000	0.08	1.000
2017 vs. 2018 Controls	0.02	1.000	0.06	1.000
2017 vs. 2019 Controls	0.08	1.000	0.06	1.000
2017 vs. 2020 Controls	0.06	1.000	0.12	0.479
2017 vs. 2021 Controls	0.03	1.000	0.12	0.479
2017 vs. 2022 Controls	0.08	1.000	0.35	**0.000**
2017 vs. 2018 Treatments	0.10	0.930	0.04	1.000
2017 vs. 2019 Treatments	0.13	0.340	0.20	**0.044**
2017 vs. 2020 Treatments	0.10	0.726	0.22	**0.014**
2017 vs. 2021 Treatments	0.15	0.275	0.21	**0.029**
2017 vs. 2022 Treatments	0.05	1.000	0.32	**0.000**

Table S5. Results of General Linear Mixed-Models (GLMM) analyses for common beetle species abundances (species with ≥ 20 specimens) in the Cerro Seco Unit 5 project site, Valles Caldera National Preserve, NM.

Family/species	Treatment effect on abundance (+/-/0)	Treatment	Year	Treatment x year	Log	Log x year	Treatment x Log	Treatment x year x log
Numerator *df*		1	5	5	1	5	1	5
Denominator *df*		10	330	330	330	330	330	330
Carabidae								
Agonum placidum								
F-value		6.48	4.55	4.21	2.57	1.72	2.59	2.61
Pr > F	Increase	**0.029**	**<.001**	**<.001**	0.110	0.130	0.108	**0.025**
Amara ellipsis								
F-value		34.90	8.68	7.62	10.18	1.89	9.92	1.90
Pr > F	Increase	**<.001**	**<.001**	**<.001**	**0.002**	0.095	**0.002**	0.093
Amara littoralis								
F-value		18.82	9.88	9.99	2.73	1.74	2.76	1.80
Pr > F	Increase	**0.001**	**<.001**	**<.001**	0.100	0.125	0.098	0.112
Calathus advena								
F-value		8.46	6.79	7.71	1.38	0.70	1.48	0.72
Pr > F	Decrease	**0.016**	**<.001**	**<.001**	0.241	0.623	0.225	0.610
Calathus opaculus								
F-value		10.51	10.28	14.56	0.40	0.70	1.92	1.60
Pr > F	Decrease	**0.009**	**<.001**	**<.001**	0.526	0.622	0.167	0.158
Carabus taedatus								
F-value		20.06	25.56	19.61	0.61	2.59	0.54	1.18
Pr > F	Decrease	**0.001**	**<.001**	**<.001**	0.436	**0.026**	0.462	0.319
Cicindela longilabris								
F-value		22.50	7.44	7.44	0.04	0.85	0.04	0.85
Pr > F	Increase	**<.001**	**<.001**	**<.001**	0.833	0.513	0.833	0.513
Cymindus cribricollis								
F-value		1.42	2.81	0.74	1.00	1.25	0.82	0.86
Pr > F	Neutral	0.234	**0.017**	0.591	0.319	0.286	0.365	0.508
Harpalus animosus								
F-value		35.63	22.99	17.19	35.49	3.68	29.03	3.17
Pr > F	Increase	**<.001**	**<.001**	**<.001**	**<.001**	**0.003**	**<.001**	**0.008**
Harpalus ellipsis								
F-value		17.68	3.46	3.81	0.06	0.64	1.52	0.39
Pr > F	Increase	**<.001**	**0.005**	**0.002**	0.805	0.668	0.218	0.854
Harpalus nigritarsis								
F-value		24.02	30.29	18.15	0.37	3.12	0.88	2.69
Pr > F	Increase	**<.001**	**<.001**	**<.001**	0.543	**0.009**	0.348	**0.021**
Harpalus somnulentus								
F-value		19.59	15.36	10.54	0.54	3.97	0.49	3.24
Pr > F	Increase	**<.001**	**<.001**	**<.001**	0.464	**0.002**	0.483	**0.007**
Notiophilus directus								
F-value		18.70	14.15	4.10	0.74	0.64	3.07	1.02
Pr > F	Decrease	**0.002**	**<.001**	**0.001**	0.390	0.668	0.080	0.406

Family/species	Treatment effect on abundance (+/-/0)	Treatment	Year	Treatment x year	Log	Log x year	Treatment x Log	Treatment x year x log
Numerator *df*		1	5	5	1	5	1	5
Denominator *df*		10	330	330	330	330	330	330
Carabidae								
Notiophilus semistriatus								
F-value		0.18	8.84	3.38	0.12	0.54	1.06	0.27
Pr > F	Neutral	0.675	**<.001**	**0.005**	0.727	0.743	0.303	0.930
Pterostichus adstrictus								
F-value		2.47	28.48	13.65	2.86	3.19	13.06	1.62
Pr > F	Decrease	0.147	**<.001**	**<.001**	0.092	**0.008**	**<.001**	0.153
Pterostichus restrictus								
F-value		26.88	49.64	9.03	3.44	2.41	15.87	2.04
Pr > F	Decrease	**<.001**	**<.001**	**<.001**	0.065	**0.036**	**<.001**	0.072
Rhadine nivalis								
F-value		13.15	6.16	5.72	0.70	4.95	2.49	2.71
Pr > F	Increase	**0.005**	**<.001**	**<.001**	0.404	**<.001**	0.116	**0.020**
Scaphinotus snowi								
F-value		0.64	6.17	5.07	2.31	1.54	1.65	1.67
Pr > F	Neutral	0.431	**<.001**	**<.001**	0.130	0.178	0.200	0.142
Sericoda bembidioides								
F-value		1.50	1.92	1.92	7.45	1.92	7.45	1.92
Pr > F	Increase in 2022	0.249	0.091	0.091	**0.007**	0.091	**0.007**	0.091
Sericoda quadripunctata								
F-value		12.77	7.90	5.80	11.56	2.72	10.17	3.11
Pr > F	Increase	**0.005**	**<.001**	**<.001**	**<.001**	**0.020**	**0.002**	**0.009**
Synuchus dubius								
F-value		0.65	14.23	2.29	0.66	0.71	0.09	5.29
Pr > F	Increase in 2021	0.439	**<.001**	**0.046**	0.416	0.614	0.764	**<.001**
Chrysomelidae								
Phyllotreta sp. #1								
F-value		3.42	3.85	1.90	0.05	0.38	0.49	0.72
Pr > F	Neutral	0.065	**0.002**	0.093	0.822	0.865	0.483	0.606
Coccinellidae								
Exochomus townsendi								
F-value		1.57	1.24	1.24	3.07	1.24	3.07	1.24
Pr > F	Neutral	0.219	0.291	0.291	0.081	0.291	0.081	0.291
Hippodamia convergens								
F-value		15.71	16.96	15.21	4.10	3.78	3.81	3.50
Pr > F	Increase	**0.003**	**<.001**	**<.001**	**0.044**	**0.002**	0.052	**0.004**
Hippodamia parenthesis								
F-value		11.64	14.36	14.39	1.94	1.34	1.74	1.35
Pr > F	Increase	**0.007**	**<.001**	**<.001**	0.165	0.248	0.189	0.242

Family/species	Treatment effect on abundance (+/-/0)	Treatment	Year	Treatment x year	Log	Log x year	Treatment x Log	Treatment x year x log
Numerator *df*		1	5	5	1	5	1	5
Denominator *df*		10	330	330	330	330	330	330
Cryptophagidae								
Atomaria sp. #1								
F-value		2.13	24.60	0.72	4.69	2.83	0.37	1.21
Pr > F	Neutral	0.175	**<.001**	0.605	**0.031**	**0.016**	0.543	0.302
Atomaria sp. #2								
F-value		11.46	14.61	11.38	2.37	1.06	2.81	0.69
Pr > F	Decrease	**0.007**	**<.001**	**<.001**	0.124	0.384	0.094	0.632
Cryptophagus pilosus								
F-value		36.84	7.02	10.43	3.93	0.64	6.24	1.06
Pr > F	Decrease	**<.001**	**<.001**	**<.001**	**0.048**	0.667	**0.013**	0.384
Cryptophagus tuberculosus								
F-value	Decrease	0.05	9.23	7.19	2.12	3.20	3.92	2.58
Pr > F	in 2020	0.836	**<.001**	**<.001**	0.147	**0.008**	**0.049**	**0.026**
Curculionidae								
Hylastes gracilis								
F-value		0.23	4.88	2.44	0.98	0.83	7.30	3.24
Pr > F	Increase in 2020	0.645	**<.001**	**0.034**	0.324	0.527	**0.007**	**0.007**
Otiorhynchus ovatus								
F-value		2.68	3.37	0.22	0.90	1.69	7.70	0.75
Pr > F	Neutral	0.133	**0.006**	0.953	0.345	0.137	**0.006**	0.587
Pseudorimus orbicollis								
F-value		0.91	2.80	2.33	0.41	0.70	2.07	1.72
Pr > F	Increase in 2019	0.363	**0.017**	**0.042**	0.525	0.622	0.151	0.129
Thricolepis inornata								
F-value		11.80	5.16	6.25	0.10	0.51	1.19	0.55
Pr > F	Decrease	**0.006**	**<.001**	**<.001**	0.750	0.772	0.276	0.735
Elateridae								
Athous arizonicus								
F-value		0.13	6.28	1.04	0.44	0.86	0.33	2.75
Pr > F	Neutral	0.723	**<.001**	0.395	0.507	0.510	0.563	**0.019**
Athous sp. #2								
F-value		0.00	13.29	0.66	4.69	1.01	0.00	0.39
Pr > F	Neutral	0.956	**<.001**	0.657	**0.031**	0.410	0.954	0.855
Limonius aeger								
F-value		1.89	0.78	3.19	0.09	1.81	0.01	1.15
Pr > F	Decrease in 2020	0.199	0.562	**0.008**	0.761	0.110	0.943	0.333
Stropenron pudica								
F-value		2.98	0.87	0.87	2.20	0.68	0.59	2.57
Pr > F	Neutral	0.095	0.499	0.499	0.139	0.640	0.442	**0.027**

Family/species	Treatment effect on abundance (+/-/0)	Treatment	Year	Treatment x year	Log	Log x year	Treatment x Log	Treatment x year x log
Numerator *df*		1	5	5	1	5	1	5
Denominator *df*		10	330	330	330	330	330	330
Elateridae								
Stropenron sp. #1								
F-value		8.74	4.03	5.39	13.11	0.70	10.74	1.12
Pr > F	Decrease	**0.014**	**0.001**	**<.001**	**<.001**	0.622	**0.001**	0.349
Endomychidae								
Aphorista morosa								
F-value		1.51	2.47	2.15	4.48	0.65	1.07	0.76
Pr > F	Neutral	0.247	**0.032**	0.059	**0.035**	0.658	0.302	0.581
Histeridae								
Hister furtivus								
F-value		0.51	0.32	1.61	6.85	0.22	0.72	1.83
Pr > F	Neutral	0.477	0.902	0.157	**0.009**	0.953	0.396	0.106
Saprinus sp. #1								
F-value		0.57	1.47	0.57	1.22	1.64	0.66	0.55
Pr > F	Neutral	0.451	0.197	0.722	0.269	0.150	0.419	0.741
Latridiidae								
Corticaria sp. #1								
F-value		3.47	15.47	5.34	3.70	0.55	10.69	1.53
Pr > F	Decrease 2021	0.092	**<.001**	**<.001**	0.055	0.737	**0.001**	0.181
Corticarina sp. #1								
F-value		2.19	2.47	1.95	0.01	1.83	0.01	0.39
Pr > F	Neutral	0.150	**0.033**	0.086	0.937	0.106	0.907	0.854
Latridius sp. #1								
F-value		4.62	2.32	3.82	1.00	1.00	0.27	1.07
Pr > F	Decrease	**0.040**	**0.043**	**0.002**	0.318	0.416	0.603	0.376
Leiodidae								
Agathidium pulchrum								
F-value		1.44	1.75	1.51	3.18	1.67	3.04	1.39
Pr > F	Neutral	0.257	0.122	0.187	0.076	0.142	0.082	0.226
Catops sp. #1								
F-value		0.04	2.30	0.21	2.90	2.63	0.58	0.30
Pr > F	Neutral	0.840	**0.045**	0.958	0.089	**0.023**	0.448	0.911
Hydnobius longidens								
F-value		2.47	1.52	1.27	2.85	1.07	1.62	0.93
Pr > F	Neutral	0.147	0.182	0.275	0.092	0.375	0.205	0.464
Ptomaphagus sp. #1								
F-value		0.13	4.38	0.20	0.03	0.05	0.39	0.37
Pr > F	Neutral	0.723	**<.001**	0.961	0.860	0.999	0.535	0.870

Family/species	Treatment effect on abundance (+/-/0)	Treatment	Year	Treatment x year	Log	Log x year	Treatment x Log	Treatment x year x log
Numerator *df*		1	5	5	1	5	1	5
Denominator *df*		10	330	330	330	330	330	330
Nitidulidae								
Epuraea alternans								
F-value		22.39	38.27	20.94	15.29	5.18	1.77	0.48
Pr > F	Decrease	**<.001**	**<.001**	**<.001**	**<.001**	**<.001**	0.184	0.788
Ptiliidae								
Acrotrichis sp. #1								
F-value		13.61	10.81	3.11	0.81	0.52	0.03	0.87
Pr > F	Decrease	**<.001**	**<.001**	**0.009**	0.368	0.765	0.856	0.503
Scarabaeidae								
Dichelonyx sp. #1								
F-value		10.67	43.42	17.30	0.09	0.15	1.33	1.17
Pr > F	Decrease	**0.008**	**<.001**	**<.001**	0.762	0.981	0.249	0.322
Scraptiidae								
Anaspis rufa								
F-value		10.48	10.55	10.86	9.65	2.49	3.73	4.66
Pr > F	Decrease	**0.009**	**<.001**	**<.001**	**0.002**	**0.031**	0.054	**<.001**
Silphidae								
Heterosilpha ramosa								
F-value		1.69	6.36	6.56	1.43	1.43	0.87	1.62
Pr > F	Increase in 2022	0.222	**<.001**	**<.001**	0.233	0.214	0.350	0.155
Nicrophorus investigator								
F-value		2.08	2.37	1.99	0.18	2.12	0.13	2.01
Pr > F	Neutral	0.150	**0.039**	0.079	0.673	0.062	0.716	0.076
Staphylinidae								
Acrolocha sp. #1								
F-value		4.78	20.28	3.35	0.11	1.58	1.10	3.60
Pr > F	Decrease in 2020	0.054	**<.001**	**0.006**	0.746	0.164	0.294	**0.003**
Acrotona sp. #1								
F-value		2.40	17.16	5.35	0.22	0.12	0.00	0.00
Pr > F	Decrease in 2020	0.152	**<.001**	**<.001**	0.636	0.989	0.960	1.000
Aleochara sp. #1								
F-value		0.16	4.01	2.28	0.74	2.37	2.11	0.63
Pr > F	Decrease in 2019	0.695	**0.002**	**0.047**	0.391	**0.039**	0.147	0.680

Family/species	Treatment effect on abundance (+/-/0)	Treatment	Year	Treatment x year	Log	Log x year	Treatment x Log	Treatment x year x log
Numerator *df*		1	5	5	1	5	1	5
Denominator *df*		10	330	330	330	330	330	330
Staphylinidae								
Aleocharinae spp.								
F-value		63.38	26.53	18.36	0.19	0.34	0.09	0.49
Pr > F	Decrease	**<.001**	**<.001**	**<.001**	0.663	0.889	0.765	0.783
Anotylus sp. #1								
F-value		2.54	15.59	3.97	0.73	0.72	0.49	0.81
Pr > F	Decrease in 2020	0.142	**<.001**	**0.002**	0.392	0.607	0.485	0.546
Belonuchus sp. #2								
F-value		5.03	2.63	1.03	0.01	0.10	0.46	0.48
Pr > F	Neutral	**0.049**	**0.024**	0.402	0.938	0.991	0.496	0.789
Bobitobus sp. #1								
F-value		1.78	10.14	10.29	0.48	0.15	0.36	0.43
Pr > F	Decrease in 2020	0.193	**<.001**	**<.001**	0.490	0.980	0.547	0.827
Bolitobius sp. #1								
F-value		44.82	18.99	15.37	1.38	2.31	0.34	3.12
Pr > F	Decrease	**<.001**	**<.001**	**<.001**	0.241	**0.044**	0.560	**0.009**
Brachycepsis sp. #1								
F-value		3.14	3.47	0.39	0.45	2.65	1.07	0.16
Pr > F	Neutral	0.077	**0.004**	0.854	0.501	**0.023**	0.301	0.977
Carpelimus sp. #1								
F-value		2.58	1.41	1.30	2.12	1.21	0.77	1.11
Pr > F	Neutral	0.139	0.221	0.262	0.147	0.306	0.382	0.353
Gabrius sp. #1								
F-value		0.17	9.65	1.61	1.00	0.26	0.04	0.21
Pr > F	Neutral	0.685	**<.001**	0.156	0.319	0.936	0.849	0.958
Lordithon sp. #1								
F-value		18.37	6.74	7.05	0.08	0.25	0.23	0.26
Pr > F	Decrease	**0.002**	**<.001**	**<.001**	0.773	0.939	0.635	0.935
Megarthrus angulicollis								
F-value		11.76	6.36	4.93	0.01	0.28	0.23	1.64
Pr > F	Decrease	**<.001**	**<.001**	**<.001**	0.912	0.922	0.629	0.148
Myrmecocephalus arizonicus								
F-value		0.77	6.79	1.21	6.22	4.01	3.74	2.07
Pr > F	Neutral	0.382	**<.001**	0.304	**0.013**	**0.001**	0.054	0.069
Oxytelus sp. #1								
F-value		1.88	2.13	1.56	1.89	0.39	1.40	0.34
Pr > F	Neutral	0.201	0.062	0.170	0.170	0.858	0.238	0.891
Palporus nitidulus								
F-value		28.45	8.01	9.15	0.34	1.11	1.16	0.90
Pr > F	Decrease	**<.001**	**<.001**	**<.001**	0.561	0.356	0.282	0.478

Family/species	Treatment effect on abundance (+/-/0)	Treatment	Year	Treatment x year	Log	Log x year	Treatment x Log	Treatment x year x log
Numerator *df*		1	5	5	1	5	1	5
Denominator *df*		10	330	330	330	330	330	330
Staphylinidae								
Philonthus sp. #1								
F-value		7.18	4.51	4.51	5.86	2.36	5.86	2.36
Pr > F	Increase	**0.023**	**<.001**	**<.001**	**0.016**	**0.040**	**0.016**	**0.040**
Phyllodrepa sp. #1								
F-value		0.26	5.21	6.40	9.80	3.97	0.27	2.48
Pr > F	Decrease in 2020	0.618	**<.001**	**<.001**	**0.002**	**0.002**	0.602	**0.032**
Phyllodrepa sp. #2								
F-value		18.26	13.13	12.21	0.85	0.51	0.59	0.51
Pr > F	Decrease	**<.001**	**<.001**	**<.001**	0.358	0.769	0.444	0.768
Proteinus sp. #1								
F-value		27.81	7.49	8.34	7.91	0.54	6.49	0.90
Pr > F	Decrease	**<.001**	**<.001**	**<.001**	**0.005**	0.748	**0.011**	0.481
Pseudopsis spp.								
F-value		4.39	5.84	5.20	8.53	1.01	1.86	1.14
Pr > F	Decrease 2019-20	0.063	**<.001**	**<.001**	**0.004**	0.410	0.173	0.339
Quedius sp. #1								
F-value		17.40	17.08	9.51	11.66	3.43	16.59	5.05
Pr > F	Decrease	**0.002**	**<.001**	**<.001**	**<.001**	**0.005**	**<.001**	**<.001**
Stenus sp. #1								
F-value		1.90	2.58	0.35	4.06	1.26	2.16	2.73
Pr > F	Neutral	0.198	**0.026**	0.881	**0.045**	0.281	0.142	**0.020**
Tachyporus sp. #1								
F-value		28.66	8.68	9.07	4.51	1.83	3.61	2.07
Pr > F	Increase	**<.001**	**<.001**	**<.001**	**0.034**	0.106	0.058	0.069
Xenota sp. #1								
F-value		9.15	11.34	8.52	2.76	2.83	2.87	2.94
Pr > F	Decrease	**0.003**	**<.001**	**<.001**	0.098	**0.016**	0.091	**0.013**
Tenebrionidae								
Eleodes carbonaria								
F-value		65.73	30.48	31.03	17.25	4.71	17.69	4.66
Pr > F	Increase	**<.001**	**<.001**	**<.001**	**<.001**	**<.001**	**<.001**	**<.001**
Eleodes snowii								
F-value		0.09	13.33	8.70	12.91	2.40	3.09	1.14
Pr > F	Neutral	0.766	**<.001**	**<.001**	**<.001**	**0.037**	0.080	0.341
Tetratomidae								
Tetratoma concolor								
F-value		4.59	11.70	8.35	0.11	0.22	1.87	0.68
Pr > F	Decrease in 2018	0.058	**<.001**	**<.001**	0.735	0.953	0.172	0.637

Family/species	Treatment effect on abundance (+/-/0)	Treatment	Year	Treatment x year	Log	Log x year	Treatment x Log	Treatment x year x log
Numerator *df*		1	5	5	1	5	1	5
Denominator *df*		10	330	330	330	330	330	330
Trachypachidae								
Trachypachus inermis								
F-value		2.37	10.55	2.48	2.87	0.85	4.92	1.15
Pr > F	Increase in 2019	0.155	**<.001**	**0.032**	0.091	0.513	**0.027**	0.331

Table S6. Model results relating habitat variables to beetle (Coleoptera) abundances (mean number/trap-year) from multiple regression analyses of the selected model ($\Delta AIC_c = 0$) in the post-treatment project area during 2018-2022, Valles Caldera National Preserve, NM. Beetles shown are species exhibiting significant abundance **increases** following treatments with ≥ 20 individuals in the samples. Variables are % basal (ground-level) cover for soil (bare), litter, rock, wood, stump, and live basal herbaceous vegetation (B-sedge, B-grass, B-forb, moss) and % canopy cover for live herbaceous canopy vegetation (C-total-herbaceous, C-sedge, C-grass, C-forb, C-nonvascular plants).

Family, Species	Model Adjusted-R^2	Model Constant (*P*-value)	Variable 1 Coefficient (*P*-value)	Variable 2 Coefficient (*P*-value)	Variable 3 Coefficient (*P*-value)	Variable 4 Coefficient (*P*-value)	Variable 5 Coefficient (*P*-value)	Variable 6 Coefficient (*P*-value)	Variable 7 Coefficient (*P*-value)
Carabidae									
Agonum placidum	0.290	-0.448<br>(0.371)	Wood<br>0.247<br>(0.003)						
Amara ellipsis	0.286	-7.940<br>(0.250)	C-grass<br>0.187<br>(0.068)	Stump<br>6.646<br>(0.011)					
Amara littoralis	0.505	29.105<br>(0.012)	B-sedge<br>3.426<br>(0.011)	Litter<br>-0.338<br>(0.018)	C-nonvas<br>-2.521<br>(0.006)				
Cicindela longilabris	0.404	-4.342<br>(0.769)	C-grass<br>1.215<br>(0.0004)						
Harpalus animosus	0.693	26.383<br>(0.073)	C-forb<br>4.609<br>(0.0000)	Rock<br>-15.943<br>(0.0003)	Wood<br>-2.593<br>(0.085)	B-forb<br>-21.058<br>(0.003)			
Harpalus littoralis	0.433	0.112<br>(0.928)	Soil<br>-0.113<br>(0.039)	B-forb<br>0.667<br>(0.047)	C-herb<br>0.177<br>(0.0004)	C-sedge<br>-0.155<br>(0.0004)	C-forb<br>-0.292<br>(0.002)		
Harpalus nigritarsis	0.461	-2.684<br>(0.687)	Soil<br>-0.507<br>(0.069)	Stump<br>4.304<br>(0.015)	C-grass<br>-0.433<br>(0.034)	C-sedge<br>-0.576<br>(0.003)	C-herb<br>0.709<br>(0.010)		
Harpalus somnulentus	0.640	-11.531<br>(0.005)	B-sedge<br>-4.208<br>(0.014)	C-forb<br>0.285<br>(0.002)	C-sedge<br>0.153<br>(0.001)	Soil<br>0.489<br>(0.011)			
Rhadine nivalis	0.313	47.174<br>(0.0000)	Stump<br>-11.608<br>(0.005)	B-sedge<br>-10.445<br>(0.116)					

Family,<br>Species	Model Adjusted-<br>R^2	Model Constant<br>(*P*-value)	Variable 1	Variable 2	Variable 3	Variable 4	Variable 5	Variable 6	Variable 7
Sericoda bembidioides	0.783	75.619<br>(0.005)	Litter<br>-0.858<br>(0.010)	Rock<br>8.699<br>(0.000)	Stump<br>-3.831<br>(0.012)	B-sedge<br>-8.210<br>(0.004)	B-moss<br>24.226<br>(0.004)	C-nonvas<br>-22.955<br>(0.0004)	
Sericoda quadripunctata	0.788	-18.169<br>(0.000)	Rock<br>4.525<br>(0.000)	Stump<br>3.954<br>(0.010)	B-sedge<br>-8.645<br>(0.000)	B-forb<br>5.618<br>(0.002)	C-herb<br>1.019<br>(0.000)	C-sedge<br>-0.785<br>(0.000)	C-forb<br>-1.700<br>(0.000)
Synuchus dubius	0.354	3.011<br>(0.162)	B-sedge<br>-5.178<br>(0.162)	C-grass<br>0.092<br>(0.029)					
Coccinellidae									
Hippodamia convergens	0.815	132.266<br>(0.000)	Litter<br>-1.458<br>(0.000)	Wood<br>-1.229<br>(0.017)	Stump<br>-4.042<br>(0.004)	B-grass<br>5.584<br>(0.004)	B-sedge<br>11.054<br>(0.001)	C-forb<br>-0.623<br>(0.004)	C-nonvas<br>-7.887<br>(0.000)
Hippodamia parenthesis	0.508	-18.230<br>(0.046)	Soil<br>0.851<br>(0.033)	Stump<br>-7.398<br>(0.004)	B-forb<br>-3.144<br>(0.041)	C-herb<br>0.828<br>(0.000)	C-grass<br>-0.784<br>(0.000)		
Hylastes gracilis	0.181	0.226<br>(0.794)	Rock<br>1.287<br>(0.019)						
Pseudorimus orbicollis	0.455	-3.887<br>(0.002)	Wood<br>0.458<br>(0.001)	C-forb<br>0.146<br>(0.004)					
Silphidae									
Heterosilpha ramosa	0.052	0.046<br>(0.956)	B-forb<br>0.912<br>(0.141)						
Staphylinidae									
Philonthus sp. #1	0.727	-2.570<br>(0.001)	B-sedge<br>1.387<br>(0.001)	B-forb<br>1.299<br>(0.000)	C-herb<br>0.193<br>(0.000)	C-sedge<br>-0.141<br>(0.000)	C-forb<br>-0.407<br>(0.000)		
Tachyporus sp. #1	0.628	1.238<br>(0.353)	Stump<br>-0.780<br>(0.085)	C-herb<br>-0.176<br>(0.016)	C-grass<br>0.153<br>(0.007)	C-sedge<br>0.186<br>(0.001)			

Family, Species	Model Adjusted-R^2	Model Constant (P-value)	Variable 1	Variable 2	Variable 3	Variable 4	Variable 5	Variable 6	Variable 7
Tenebrionidae									
Eleodes carbonaria	0.906	-25.835 (0.000)	B-sedge<br>-7.020<br>(0.016)	B-grass<br>-11.972<br>(0.000)	C-sedge<br>-0.790<br>(0.000)	C-forb<br>1.296<br>(0.000)	C-nonvas<br>9.247<br>(0.001)		
Trachypachidae									
Trachypachus inermis	0.181	2.372 (0.689)	B-sedge<br>22.472<br>(0.020)						

Table S7. Model results relating habitat variables to beetle (Coleoptera) abundances (mean number/trap-year) from multiple regression analyses of the selected model ($\Delta AIC_c = 0$) in the post-treatment project area during 2018-2022, Valles Caldera National Preserve, NM. Beetles shown are species exhibiting significant abundance **decreases** following treatments with ≥ 20 individuals in the samples. Variables are % basal (ground-level) cover for soil (bare), litter, rock, wood, stump, and live basal herbaceous vegetation (B-sedge, B-grass, B-forb, moss) and % canopy cover for live herbaceous canopy vegetation (C-total-herbaceous, C-sedge, C-grass, C-forb, C-nonvascular plants).

Family, Species	Model Adjusted-R^2	Model Constant (*P*-value)	Variable 1 Coefficient (*P*-value)	Variable 2 Coefficient (*P*-value)	Variable 3 Coefficient (*P*-value)	Variable 4 Coefficient (*P*-value)	Variable 5 Coefficient (*P*-value)	Variable 6 Coefficient (*P*-value)	Variable 7 Coefficient (*P*-value)
Carabidae									
Calathus advena	0.287	4.456 (0.003)	Rock -0.993 (0.042)	Wood -0.567 (0.006)					
Calathus opaculus	0.444	4.174 (0.009)	B-sedge -3.656 (0.064)	B-forb 3.192 (0.001)	Moss -15.007 (0.004)	C-nonvas 11.184 (0.002)			
Carabus taedatus	0.437	35.354 (0.320)	Rock -22.822 (0.008)	B-grass 31.029 (0.020)	B-sedge -69.574 (0.004)	Moss -145.638 (0.017)	C-sedge 1.617 (0.018)	C-nonvas 31.029 (0.020)	
Notiophilus directus	0.444	0.870 (0.142)	Soil -0.071 (0.111)	B-forb 0.776 (0.000)					
Pterostichus adstrictus	0.636	-6.945 (0.729)	Rock -12.337 (0.017)	Stump 20.469 (0.007)	Moss -84.978 (0.018)	C-forb 3.962 (0.000)	C-nonvas 73.646 (0.004)		
Pterostichus restrictus	0.649	55.823 (0.000)	Rock -4.909 (0.001)	B-grass 12.568 (0.000)	C-herb -0.544 (0.000)				
Cryptophagidae									
Atomaria sp. #2	0.547	-11.856 (0.000)	Litter 0.159 (0.000)	Stump 0.535 (0.018)	B-forb 0.384 (0.030)	C-herb 0.075 0.034)	C-grass -0.067 (0.011)	C-sedge -0.072 (0.007)	
Cryptophagus pilosus	0.271	0.360 (0.702)	Wood 0.448 (0.004)						

Family,<br>Species	Model Adjusted-R^2	Model Constant (P-value)	Variable 1	Variable 2	Variable 3	Variable 4	Variable 5	Variable 6	Variable 7
Latridiidae									
Corticaria sp. #1	0.658	41.146<br>(0.000)	B-sedge<br>26.456<br>(0.000)	Moss<br>51.031<br>(0.002)	C-grass<br>-0.447<br>0.006	C-nonvas<br>-37.605<br>(0.001)			
Latridius sp. #1	0.437	-0.355<br>(0.714)	B-forb<br>-1.767<br>(0.003)	C-grass<br>-0.157<br>(0.002)	C-sedge<br>0.083<br>(0.001)	C-forb<br>0.400<br>(0.003)			
Nitidulidae									
Epuraea alternans	0.764	5.577<br>(0.022)	Moss<br>-13.216<br>(0.000)	C-herb<br>-0.060<br>(0.028)	C-nonvas<br>9.782<br>(0.000)				
Scarabaeidae									
Dichelonyx sp. #1	0.382	-17.600<br>(0.007)	Litter<br>0.223<br>(0.011)	Stump<br>1.779<br>(0.002	C-herb<br>0.066<br>(0.027)	C-sedge<br>-0.139<br>(0.001)			
Staphylinidae									
Acrotona sp. #1	0.682	0.600<br>(0.261)	B-grass<br>1.082<br>(0.002)	C-grass<br>-0.084<br>(0.000)	C-forb<br>0.183<br>(0.002)				
Aleochara sp. #1	0.234	0.185<br>(0.741)	Wood<br>0.231<br>(0.008)	B-grass<br>-0.557<br>(0.096)					
Aleocharinae spp.	0.463	-27.543<br>(0.044)	Rock<br>8.307<br>(0.015)	B-forb<br>18.209<br>(0.010)	Moss<br>19.710<br>(0.037)	C-grass<br>1.618<br>(0.004)	C-forb<br>-3.206<br>(0.030)		
Anotylus sp. #1	0.364	4.167<br>(0.142)	Rock<br>1.803<br>(0.005)	Stump<br>2.121<br>(0.015)	Moss<br>8.328<br>(0.047)	C-sedge<br>-0.130<br>(0.012)	C-nonvas<br>-7.804<br>(0.015)		
Palporus nitidulus	0.761	-5.856<br>(0.002)	Wood<br>0.358<br>(0.068)	B-forb<br>1.150<br>(0.002)	Moss<br>1.298<br>(0.044)	C-herb<br>0.200<br>(0.000)	C-sedge<br>-0.126<br>(0.001)	C-forb<br>-0.360<br>(0.000)	

References

Parmenter, RR, Losleben, MV. 2023. Influence of mixed conifer forest thinning and prescribed fire on soil temperature and moisture dynamics in proximity to forest logs: A case study in New Mexico, USA. Forests 14, 1117. https://doi.org/10.3390/f14061117

Parmenter, RR, Obermeit, TJ, Stout, BP, et al. 2026. Forest restoration thinning and burning treatments create habitat mosaics and facilitate succession of grasshopper and cricket communities (Orthoptera). For. Ecol. Manage. 607:123576. https://doi.org/10.1016/j.foreco.2026.123576
